# Identification of long non-coding RNA-microRNA-mRNA regulatory modules and their potential roles in drought stress response in wheat (*Triticum aestivum* L.)

**DOI:** 10.3389/fpls.2022.1011064

**Published:** 2022-10-11

**Authors:** Ning Li, Tongtong Liu, Feng Guo, Jinwen Yang, Yugang Shi, Shuguang Wang, Daizhen Sun

**Affiliations:** College of Agronomy, Shanxi Agricultural University, Jin Zhong, China

**Keywords:** wheat, drought stress, long noncoding RNA, micro RNA, mRNA

## Abstract

Drought is one of the most severe abiotic stresses that influence wheat production across the globe. Understanding the molecular regulatory network of wheat in response to drought is of great importance in molecular breeding. Noncoding RNAs influence plant development and resistance to abiotic stresses by regulating gene expression. In this study, whole-transcriptome sequencing was performed on the seedlings of two wheat varieties with contrasting levels of drought tolerance under drought and control conditions to identify long noncoding RNAs (lncRNAs), micro RNAs (miRNAs), and mRNAs related to drought stress and explore the potential lncRNA-miRNA-mRNA regulatory modules in controlling wheat drought stress response. A total of 1515 differentially expressed lncRNAs (DELs), 209 differentially expressed miRNAs (DEMs), and 20462 differentially expressed genes (DEGs) were identified. Of the 20462 DEGs, 1025 were identified as potential wheat drought resistance-related DEGs. Based on the regulatory relationship and expression patterns of DELs, DEMs, and DEGs, 10 DEL-DEM-DEG regulatory modules related to wheat drought stress response were screened, and preliminary expression verification of two important candidate modules was performed. Our results revealed the possible roles of lncRNA-miRNA-mRNA modules in regulatory networks related to drought tolerance and provided useful information as valuable genomic resources in molecular breeding of wheat.

## Introduction

Drought stress is one of the major agricultural risks, and improving drought-tolerant crops is an urgent need in China and around the world (Araus et al., 2008). Wheat (*Triticum aestivum* L.) is one of the three major food crops in the world. Mining its candidate genes in response to drought stress and analyzing its genetic regulatory basis can provide new gene resources and a genetic basis for molecular design breeding of drought-tolerant wheat. Noncoding RNAs (ncRNAs) are a class of RNA molecules that do not encode proteins and have catalytic activity, and are widely present in various organisms (Mattick, 2009). Based on structure, ncRNAs can be divided into linear ncRNAs (linear-ncRNAs) and circular ncRNAs (circRNAs). Linear ncRNAs include microRNAs (miRNAs) and long noncoding RNAs (lncRNAs) (Mattick, 2005). In recent years, studies have found that ncRNAs, especially miRNAs and lncRNAs, can regulate gene expression at the gene transcriptional and protein translational levels and participate in almost all important life processes such as development, differentiation, and metabolism (Wang and Chang, 2011; Zou et al., 2020).

MiRNAs negatively regulate the expression of target genes at the post-transcriptional level, and their important roles in plant growth and development, stress response, and other physiological and biochemical reactions have been verified (Carrington and Ambros, 2003; Khraiwesh et al., 2012). In addition, lncRNAs can competitively bind with miRNAs to coordinately regulate the expression of target genes, and thereby life processes (Guttman and Rinn, 2012; Xu et al., 2020). Recent studies have identified a large number of lncRNAs and miRNAs related to growth and development and abiotic stress response in wheat. Lu et al. (2020b) found that 7435 lncRNAs were co-expressed with 7191 mRNAs, and 6 lncRNA-mRNA modules associated with cold stress response in wheat were obtained. Using two pairs of near-isogenic lines, Zhou et al. (2020) identified 5399 tillering-associated lncRNAs in wheat. Cao et al. (2021) performed genome-wide profiling of lncRNAs during wheat spike development in 6 different stages and identified a total of 8889 expressed lncRNAs, among which 2753 were differentially expressed in various developmental stages. Akdogan et al. (2016) investigated drought stress-responsive miRNAs in the root and leaf tissues of bread wheat. Their results showed that 285 and 244 miRNAs were differentially expressed in leaf and root tissues, respectively. A total of 79 miRNAs (46 known and 33 novel miRNAs) that showed significant differential expression during wheat grain development under both high- and low-nitrogen treatments were identified (Hou et al., 2020). Under cold stress, a total of 192 miRNAs from 105 families and nine novel miRNAs were identified. Among them, 34 conserved and five novel miRNAs were differentially expressed between cold-stressed samples and controls (Song et al., 2017).

At present, research on ncRNAs related to drought stress tolerance in wheat mostly focuses on the combined analyses of single ncRNAs and mRNAs (Lu et al., 2020a; Madhawan et al., 2020; Wu et al., 2021), with no reports available on the combined analyses of lncRNAs, miRNAs, and mRNAs. Therefore, this study aims to explore the role of lncRNAs, miRNAs, and mRNAs in wheat drought resistance, and identify differentially expressed lncRNAs, miRNAs, and genes for the same. Based on the expression patterns of ncRNAs and mRNAs, lncRNA-miRNA-mRNA regulatory modules related to wheat drought stress response were screened, and preliminary expression verification of important candidate modules was performed. The results of this study will provide a basis for the next step analysis of the regulatory functions among lncRNAs, miRNAs, and genes related to drought resistance in wheat.

## Material and methods

### Plant material and drought treatment

Two wheat (*Triticum aestivum* L.) varieties, ZM13 and JM38, were used in this study. ZM13 is a drought-tolerant variety (marked as DT), and JM38 is a drought-sensitive variety (marked as DS). Wheat seeds were sterilized with 75% (v/v) ethanol for 2 min and 2% (v/v) H_2_O_2_ for 30 min, followed by washing thrice with sterile water (Quan et al., 2016). The seeds were then germinated in distilled water at room temperature. After 3 days, the germinated seeds were transplanted into 96-well plastic boxes (13cm×8.5cm×11cm) and cultivated in a light incubator at 22/20 ˚C with a 14h light/10h dark photoperiod (Sun et al., 2020). For the drought treatment of wheat seedlings, 7-day-old (one-leaf-one-heart period) wheat seedlings were cultured in Hoagland nutrient solution with or without 20% PEG 6000.

### Measurement of phenotypic and physiological traits

Relative water content (RWC) of leaf and physiological traits were measured after drought stress for four days. The last fully expanded leaves were sampled to measure RWC according to the Barrs and Weatherley method (Barrs and Weatherley, 1962). Malondialdehyde (MDA) content was measured as previously described (Cao et al., 2009). Peroxidase (POD) activity was determined as described previously (Chen et al., 2013). Proline content was measured according to the Qin et al. (2014) method. Plant height and root length were measured after drought stress for 7 days.

### Total RNA extraction, library construction, and sequencing

Total RNA was extracted from wheat root samples after four days of drought treatment using a TruSeq Stranded Total RNA Sample Preparation kit (Illumina, San Diego, USA). The RNA concentration and purity were assessed by a NanoDrop2000 (NanoDrop, Wilmington, DE, USA). The RNA integrity was assessed using both gel electrophoresis and the Agilent Bioanalyzer 2100 System (Agilent, Palo Alto, CA, USA). The ribosomal RNA (rRNA) was removed from total RNA with a Ribo-Zero™ rRNA Removal Kit (Illumina, San Diego, USA).

For lncRNA-seq, RNA was fragmented into small pieces and the first-strand cDNA synthesized with SuperScript II Reverse Transcription (Invitrogen, CA, USA). After purification, the second-strand cDNA library was synthesized following several rounds of PCR amplification. RNA library was sequenced on an Illumina NovaSeq 6000 platform at Biomarker Technologies Co., Ltd. (Beijing, China) with paired-end reads being generated.

For small RNA sequencing, total RNA was ligated to 5’ and 3’ RNA adaptors according to the manual of NEBNext Multiplex Small RNA Library Prep Set for Illumina (NEB, MA, USA). RNAs were reverse transcribed to cDNAs, following PCR amplification. PAGE gel was used to electrophoresis fragment screening purposes and rubber cutting recycling as the pieces get small RNA libraries. PCR products were then purified (AMPure XP system) and library quality assessed. The clustering of the index-coded samples was performed on a cBot Cluster Generation System using TruSeq PE Cluster Kit v4-cBot-HS (Illumia) according to the manufacturer’s instructions. After cluster generation, the libraries were sequenced by Biomarker Technologies Co., Ltd. (Beijing, China) on an Illumina NovaSeq 6000 platform with single-end reads being generated.

All raw read sequences were uploaded to the Sequence Read Archive (SRA) of NCBI under the accession numbers PRJNA838787 (lncRNA and mRNA) and PRJNA837867 (miRNA), respectively.

### lncRNAs identification and prediction of target genes

Clean data (clean reads) were obtained by first trimming adapters, and then removing reads containing ploy-N and low-quality reads from raw data with Cutadapt. Simultaneously, Q20, Q30, GC-content, and sequence duplication levels of the clean data were calculated (Ewing et al., 1998). The transcriptome was then assembled using the StringTie (1.3.1) based on the reads mapped to the reference genome using HISAT (2.0.4) (Pertea et al., 2016). The assembled transcripts were annotated using the GffCompare program. Unknown transcripts were used to screen for putative lncRNAs. Four computational approaches, including CPC (Coding Potential Calculator) (Kong et al., 2007), CNCI (Coding-Non-Coding Index) (Sun et al, 2013), Pfam (Protein family) (Finn et al., 2013), and CPAT (Coding Potential Assessment Tool) (Wang et al., 2013) were combined to screen RNA with no coding function from transcripts longer 200 nucleotides (nt) and with exon number greater than two (Kelley and Rinn, 2012). Different types of lncRNAs, including long intergenic non-coding RNA (lincRNA), intronic lncRNA, anti-sense lncRNA, and sense lncRNA were selected using Cuffcompare.

Based on the mode of action of lncRNAs and their target genes, two prediction methods were adopted. First, a Perl script was used to set the adjacent genes within 100 kb upstream and downstream of lncRNA as its cis-target genes. In addition, the trans-target genes of lncRNAs were predicted by the correlation analysis method between the expression levels of lncRNAs and mRNAs in the samples. Pearson correlation coefficient method was used to analyze the correlation between lncRNAs and mRNAs in the samples, and the gene with an absolute correlation value greater than 0.9 and *p*-value less than 0.01 was taken as the trans-target gene of lncRNA.

### miRNAs identification and prediction of target genes

First, clean data (clean reads) were obtained by first trimming adapters, and then removing reads containing ploy-N and low-quality reads from raw data. Reads were trimmed and cleaned by removing sequences smaller than 18 nt or longer than 30 nt. Simultaneously, Q20, Q30, GC-content, and sequence duplication levels of the clean data were calculated. All downstream analyses were based on clean data with high quality. Use Bowtie tools soft, the clean reads respectively with Silva database, GtRNAdb database, Rfam database, and Repbase database sequence alignment, filter ribosomal RNA (rRNA), transfer RNA (tRNA), small nuclear RNA (snRNA), small nucleolar RNA (snoRNA), and other ncRNA and repeats, respectively (Langmead et al., 2009). The remaining reads were used to detect known miRNAs by comparing with them genome and known miRNAs from miRBase release 22 (http://www.mirbase.org). MiRDeep2 software was used to predict novel miRNAs (Friedlander et al., 2012). TargetFinder software was used based on sequence information to predict miRNA target genes (Allen et al., 2005).

### Differential expression analysis and gene annotation

StringTie (1.3.1) (Trapnell et al., 2010) was used to calculate FPKMs (Fragments Per Kilobase of transcript per Million fragments mapped) of lncRNAs and mRNAs in each sample. TPM algorithm was used to normalize the expression levels of miRNAs in each sample (Li et al., 2009). DESeq R package (1.10.1) was used to analyze the differential expression levels of two groups (Love et al., 2014). The criteria to identify differentially expressed genes (DEGs), differentially expressed lncRNAs (DELs) and differentially expressed miRNAs (DEMs) were adjusted to *p*-value < 0.05 and absolute value log_2_ (fold change) > 1.

Gene Ontology (GO) enrichment analysis of the DEGs was implemented by the topGO R package (Ashburner et al., 2000). KOBAS software was used to test the statistical enrichment of DEGs in KEGG (Kyoto Encyclopedia of Genes and Genomes) pathways (Kanehisa et al., 2004).

### Quantitative real-time PCR (qRT-PCR) analysis

Quick RNA isolation Kit (Tiangen Biochemical Technology Co., Ltd., Beijing, China) was used to extract RNA according to the manufacturer’s instructions and DNase I treatment was used to remove DNA contamination. Synthesis of the first strand of cDNA was carried out according to the instructions of the kit (TaKaRa, Beijing, China). mRNA and lncRNA were detected by TB Green Premix Ex Taq II (TliRNaseH Plus) and ROX plus (TaKaRa, Beijing, China). The first-strand cDNA synthesis reagent of miRNA was performed simultaneously by A addition method and reverse transcription reaction, and NovoScript^®^ miRNA First-Strand cDNA Synthesis and SYBR qPCR Kit (Suzhou Nearshore Protein Technology Co., Ltd., Suzhou, China) was used. qRT-PCR reactions were conducted using the following protocol: 95 ˚C for 2 min, followed by 40 cycles of 95 ˚C for 20 s and 60 ˚C for 20 s and 72 ˚C for 20 s. The wheat elongation factor (*TaEF-1a*) gene was used as an internal reference for mRNAs and lncRNAs expression analyses. miRNAs expression analyses using *TaU6* gene as an internal reference gene. Threshold values (CT) were generated using the ABI PRISM 7500 system (Applied Biosystems, Foster City, CA, USA), and the transcript levels assessed using the comparative 2^-ΔΔCT^ method. All primers used in this study are listed in **supplementary Table S1**.

## Results

### Phenotypic and physiological responses of the two wheat varieties to drought stress at the seedling stage

**Figure 1:**
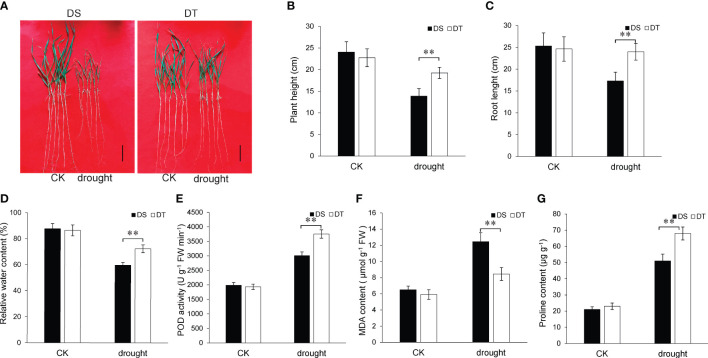
Phenotypes of the two wheat varieties under drought stress and control conditions. **(A)** Phenotypes of the two wheat varieties at the seedling stage under drought stress and control conditions for seven days. DS, drought-sensitive variety JM38, DT, drought-tolerant variety ZM13; Scale bars, 5 cm. **(B)** Height of plants subjected to drought stress for seven days. Values are means ± SD. **P < 0.01. **(C)** Root length in plants subjected to drought stress for seven days. **(D)** Relative water content of plant leaves subjected to drought stress for four days. **(E)** Peroxidase activity of plant leaves subjected to drought stress for four days. **(F)** Malindialdehyde content of plant leaves subjected to drought stress for four days. **(G)** Proline content of plant leaves subjected to drought stress for four days.

As observed in **Figure 1**, drought stress inhibited plant height and root length after 7 days of treatment. However, the plant height and root length of DT were significantly higher compared to those of DS after 7 days of drought stress (**Figures 1A–C**). Physiological traits of the two wheat varieties were measured after four days of drought stress. The results showed that DT had higher leaf RWC, proline content, and POD activity but lower MDA content compared to DS under drought stress conditions (**Figures 1D–G**). The above results proved that DT had a stronger ability to resist drought stress than DS.

### Sequencing statistics

Roots of the two wheat varieties were collected as three biological replicates at the seedling stage under drought and control conditions. A total of 210.36 Gb clean data were obtained after filtering raw data obtained by the sequencing platform. For each sample, the clean data were higher than 14.50 Gb with Q30 ≥ 93.01% (**Table 1**). Clean reads of each sample were aligned with the wheat reference genome, and alignment efficiency ranged from 71.35 to 82.74% (**Table 1**). The coding potential of the transcript was screened by the most widely used coding potential analysis methods, including CPC analysis, CNCI analysis, CPAT analysis, and Pfam protein domain analysis, to determine whether the transcript was lncRNA or not. The noncoding transcripts identified by the four analysis methods were intersected, and 15307 lncRNAs were identified (**Supplementary Figure S1**), including 13360 (87.3%) lincRNAs, 935 (6.1%) intronic lncRNAs, 680 (4.4%) antisense lncRNAs, and 332 (2.2%) sense lncRNAs (**Supplementary Figure S2**). The identified lncRNAs were subjected to subsequent analyses.

**Table 1:** Statistical table of sample sequencing data.

Sample ID^a^	lncRNA-seq			small RNA-seq		
	Clean reads	Q30 (%)^b^	Mapped reads	Clean reads	Q30 (%)	Mapped Reads
DSCK1	118952370	93.08	94119777 (79.12%)	20197784	96.29	2515305 (34.34%)
DSCK2	116572800	93.19	86353396 (74.08%)	23358263	96.29	2500611 (27.67%)
DSCK3	120557430	93.60	97212123 (80.64%)	21992366	96.22	2401561 (28.86%)
DST1	97797440	94.01	69777578 (71.35%)	20419647	96.23	924997 (21.99%)
DST2	115322786	94.79	92993676 (80.64%)	21702690	96.35	1706282 (30.71%)
DST3	101091222	93.24	82925187 (82.03%)	20658685	95.88	790809 (18.22%)
DTCK1	109456900	95.37	90563025 (82.74%)	24152317	96.93	2853912 (27.82%)
DTCK2	158882218	94.23	121387134 (76.40%)	21994070	96.84	2597988 (32.60%)
DTCK3	129129306	93.53	94179508 (72.93%)	20976188	96.68	2748927 (32.46%)
DTT1	120213128	93.34	96027095 (79.88%)	21784825	95.06	1127065 (18.02%)
DTT2	106276928	93.01	85659570 (80.60%)	21455827	96.72	1284321 (28.42%)
DTT3	119369138	93.18	94119346 (78.85%)	22729028	96.88	1542218 (33.69%)

a DSCK1-3, samples of drought-sensitive wheat varieties under control conditions; DST1-3, samples of drought-sensitive wheat varieties under drought conditions; DTCK1-3 samples of drought-tolerant wheat varieties under control conditions; DTT1-3, samples of drought-tolerant wheat varieties under drought conditions.

b Q30, percentage of bases with quality value greater than or equal to Q30 in clean data.

To understand drought-responsive and tolerance-related miRNAs in wheat, small RNA libraries of 12 samples were sequenced. In total, 375.98 million raw short reads were obtained. A total of 261.42 million clean reads were acquired after filtering out reads with low-quality and substandard sequence lengths (fewer than 18 nt or greater than 30 nt). For each sample, the clean reads were higher than 20.20 million. Q30 scores of all samples ranged from 95.06 to 96.93%, indicating that the sequence data were of reliable quality (**Table 1**). rRNA, tRNA, snRNA, snoRNA, and repetitive sequences were filtered out from the clean reads to obtain unannotated reads. The unannotated reads were aligned with the wheat reference genome, and the percentage of mapped reads in each sample’s unannotated reads ranged from 18.02 to 34.34% (**Table 1**). It was found that 93 known miRNAs and 779 novel miRNAs were expressed (**Supplementary Table S2**). In addition, the length distribution of the expressed known miRNAs was highly enriched in 21 and 22 nt and for novel miRNAs in 24 and 21 nt. (**Supplementary Figure S3**).

### Identification of DEGs

**Figure 2:**
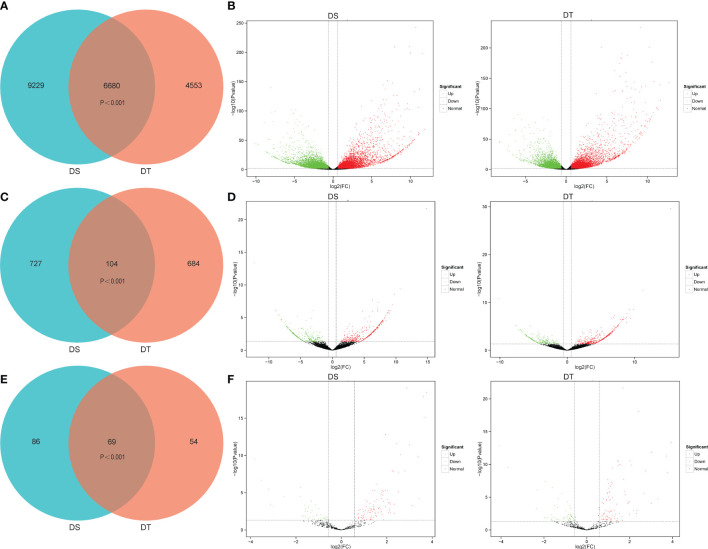
Differentially expressed genes (DEGs), differentially expressed lncRNAs (DELs), and differentially expressed miRNAs (DEMs) at the seedling stage of the two wheat varieties under drought stress relative to control conditions. DS, drought-sensitive variety JM38, DT, drought-tolerant variety ZM13. **(A, B)** Venn diagram and volcano plots of DEGs of JM38 (DS) and ZM13 (DT). **(C, D)** Venn diagram and volcano plots of DELs of JM38 (DS) and ZM13 (DT). **(E, F)** Venn diagram and volcano plots of DEMs of JM38 (DS) and ZM13 (DT).

To investigate the expression profiles of genes present in wheat roots in response to drought stress, expression levels were compared based on the FPKM values. In total, 15909 differentially expressed genes (DEGs) were identified in DS under drought conditions compared to control conditions (CK), including 6733 up-regulated and 9176 down-regulated genes, respectively. A total of 11233 DEGs were identified in DT following drought treatment compared to CK, including 5488 up-regulated and 5745 down-regulated genes, respectively. Venn diagram analysis showed that 6680 DEGs were commonly identified between DS and DT. In addition, 9229 and 4553 DEGs were specific to DS and DT, respectively (**Figures 2A, B**), and a total of 20462 DEGs were identified in both varieties.

### Screening and functional annotation of drought resistance-related DEGs

**Figure 3:**
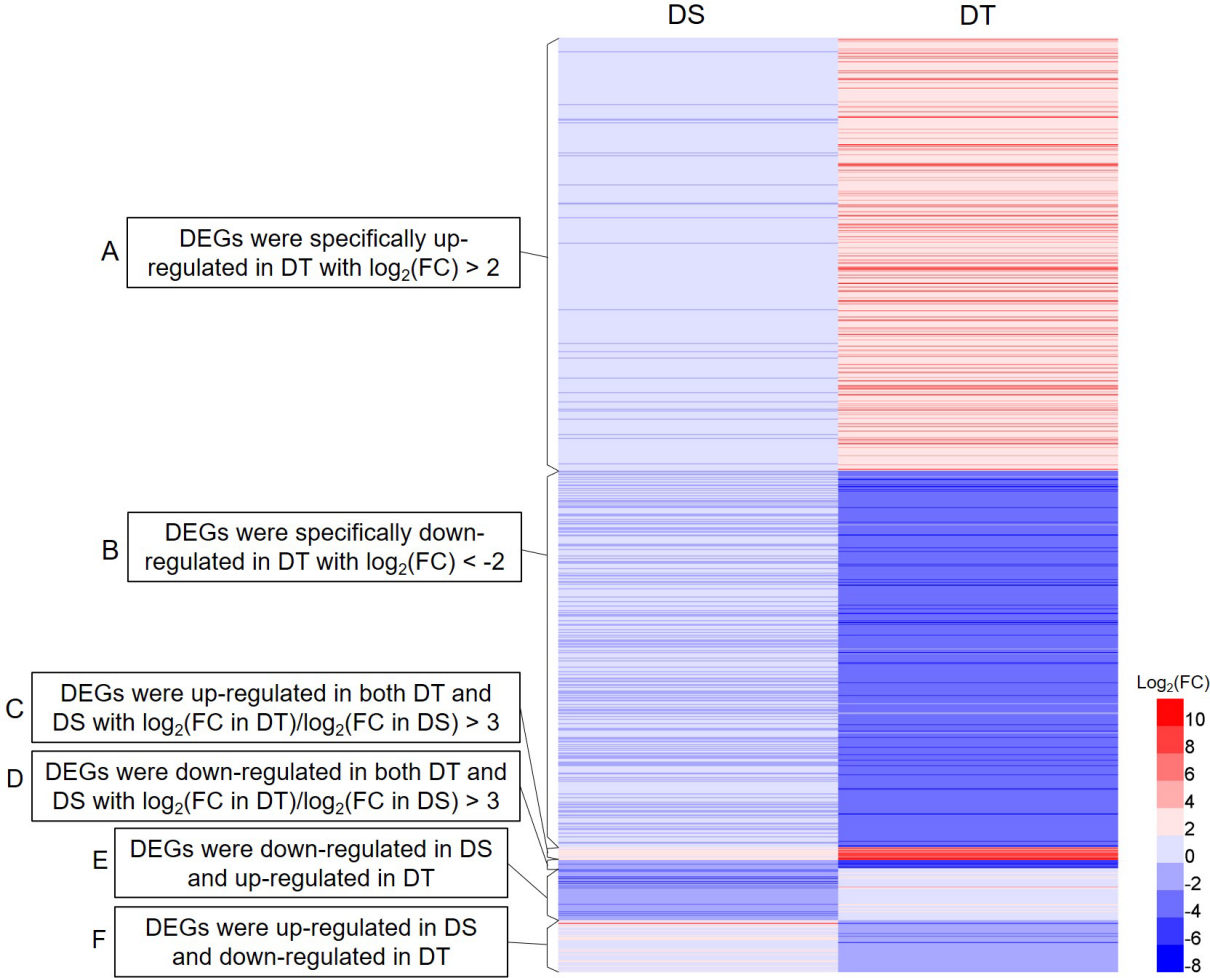
Heatmap of the expression of 1025 drought resistance-related DEGs. DS, drought-sensitive variety JM38, DT, drought-tolerant variety ZM13. **(A)** DEGs were specifically up-regulated in DT with log_2_ (fold change in DT) > 2. **(B)** DEGs were specifically down-regulated in DT with log_2_ (fold change in DT) < -2. **(C)** DEGs were up-regulated in both DT and DS with log_2_(fold change in DT)/log_2_(fold change in DS) > 3. **(D)** DEGs were down-regulated in both DT and DS with log_2_(fold change in DT)/log_2_(fold change in DS) > 3. **(E)** DEGs were down-regulated in DS and up-regulated in DT. **(F)** DEGs were up-regulated in DS and down-regulated in DT.

Of all DEGs, the following three types were screened as drought resistance-related DEGs for further analyses. The first type of DEGs were specifically up- or down-regulated in DT with an absolute value of log_2_(fold change in DT) > 2. The second type of DEGs were up- or down-regulated in both DT and DS with log_2_(fold change in DT)/log_2_(fold change in DS) > 3. The third type of DEGs showed opposite expression patterns in DT and DS. Finally, a total of 1025 drought resistance-related DEGs were screened from 20462 DEGs (**Figure 3**; **Supplementary Table S3**). To verify the accuracy of the RNA-seq data, 12 drought resistance-related DEGs were randomly selected for further qRT-PCR. The qRT-PCR and RNA-seq findings were highly consistent, indicating the reliability of the mRNA data (**Supplementary Figure S4**).

GO enrichment analysis was performed on the 1025 drought resistance-related DEGs. A total of 751 DEGs were assigned into three categories, namely biological processes (BP), cellular components (CC), and molecular functions (MF). GO terms metallic, cellular, and single organic processes were significantly enriched in BP. The significantly enriched GO terms in MF included catalytic activity, binding, and transcription factor activity, and those in CC included membrane, membrane part, and cell (**Supplementary Figure S5**). For all drought resistance-related DEGs, a total of 394 DEGs were mapped to different KEGG pathways. The results showed that pathways, such as nitrogen metabolism, alanine, aspartate, and glutamate metabolism, diterpenoid biosynthesis, phenylpropanoid biosynthesis, betalain biosynthesis, and glycerophospholipid metabolism, were significantly enriched (**Supplementary Figure S6**). In addition, pathways, including plant-pathogen interaction, phenylpropanoid biosynthesis, plant hormone signal transduction, starch and sucrose metabolism, and flavonoid biosynthesis, were enriched with more DEGs. (**Supplementary Figure S6**).

### Drought resistance-related DEGs involved in important pathways

**Figure 4:**
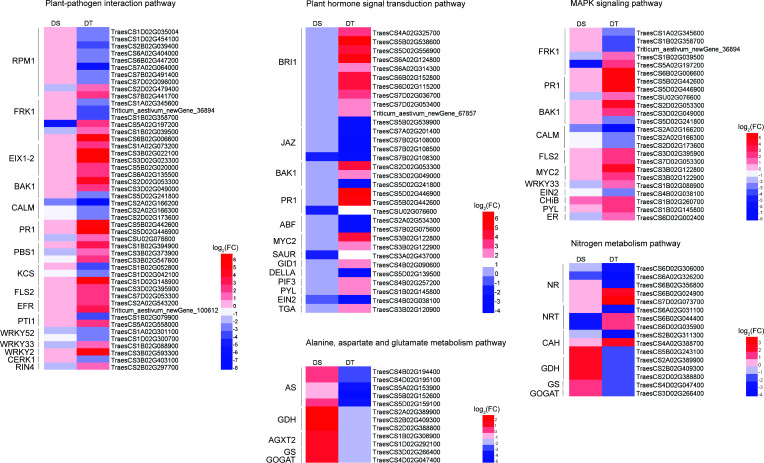
Heatmap of the expression of drought-resistant DEGs involved in important pathways. DS, drought-sensitive variety JM38, DT, drought-tolerant variety ZM13, RPM1, disease resistance protein RPM1, FRK1, serine/threonine-protein kinase FRK1, EIX 1-2, ethylene-inducing xylanase receptor, BAK1, brassinosteroid insensitive 1-associated receptor kinase 1, CALM, calmodulins, PR1, pathogenesis-related protein 1, PBS1, serine/threonine-protein kinase PBS1, KCS, 3-ketoacyl-CoA synthase, FLS2, serine/threonine-protein kinase FLS2, EFR, serine/threonine-protein kinase EFR, PTI1, pto-interacting protein 1, WRKY52, WRKY transcription factor 52, WRKY33, WRKY transcription factor 33, WRKY2, WRKY transcription factor 2, CERK1, chitin elicitor receptor kinase 1, RIN4, RPM1-interacting protein 4, BRI1, brassinosteroid insensitive 1, JAZ, jasmonate ZIM domain-containing protein, ABF, ABA-responsive element binding factor, MYC2, transcription factor MYC2, SAUR, SAUR family protein, GID1, gibberellin receptor GID1, gibberellin receptor GID1, PIF3, phytochrome-interacting factor 3, PYL, abscisic acid receptor PYL, EIN2, ethylene-insensitive protein 2, TGA, transcription factor TGA, CHiB endochitinase B, ER, serine/threonine-protein kinase ERECTA, NR, nitrate reductase, NRT, nitrate transporter, CAH, carbonic anhydrase, GDH, glutamate dehydrogenase, GS, glutamine synthetase, GOGAT, glutamate synthase, AS, Asparagine synthase, AGXT2, alanine-glyoxylate transaminase.

When plant cells are stimulated by the outside environments, they can transmit signals into the cell by activating the plant-pathogen interaction pathway, and then activate a series of other proteins (such as protein kinases) to transmit these signals (Li et al., 2020). In the present study, 48 drought resistance-related DEGs were enriched in the plant-pathogen interaction pathway, including ten disease resistance proteins RPM1, six serine/threonine-protein kinases FRK1, five EIX (ethylene-inducing xylanase) receptors, three brassinosteroid insensitive 1-associated receptor kinase 1 (BAK1), three calmodulins (CALM), three serine/threonine-protein kinases PBS1, and three pathogenesis-related protein 1 (PR1), etc. (**Figure 4**). The mitogen-activated protein kinases (MAPK) signaling pathway is one of the important pathways in the eukaryotic signaling network, which can coordinate cellular responses to achieve normal plant growth and development, immune responses, and responses to abiotic stress (Danquah et al., 2014). A total of 24 drought resistance-related DEGs, including two serine/threonine-protein kinases FLS2, four WRKY transcription factors, two transcription factor MYC2, and one abscisic acid receptor PYL were enriched in the MAPK signaling pathway (**Figure 4**). External environmental stress will stimulate the production of phytohormones and promote the plant adaptation to stressful environment (Yu et al., 2020). Here, 32 drought resistance-related DEGs were found to be enriched in the plant hormone signal transduction pathway, including four jasmonate ZIM domain-containing proteins (JAZ), two ABA-responsive element binding factors (ABF), one SAUR family protein, one ethylene-insensitive protein 2 (EIN2) and one transcription factor TGA (**Figure 4**). In addition, we found that many drought resistance-related DEGs were involved in multiple pathways, such as BAK1 and PR1 were simultaneously involved in the plant-pathogen interaction pathway, MAPK signaling pathway, and plant hormone signal transduction pathway. FLS2, WRKY transcription factor 33 (WRKY33), and FRK1 were also involved in the plant-pathogen interaction pathway and MAPK signaling pathway. PYL, EIN2, and MYC2 were involved in both MAPK signaling pathway and plant hormone signal transduction pathway (**Figure 4**). These genes might be promoted the production of phytohormones by participating in the transduction of stimulatory signals to improve the drought resistance of wheat.

In addition, we also identified 5 drought resistance-related DEGs that were enriched in the glutamate metabolism pathway and nitrogen metabolism pathway, including three glutamate dehydrogenases (GDH), one glutamine synthetase (GS), and one glutamate synthase (GOGAT) (**Figure 4**). In addition to these DEGs, 11 drought resistance-related DEGs including five nitrate reductases (NR), three nitrate transporters (NRT), and three carbonic anhydrases (CAH) were enriched in the nitrogen metabolism pathway, of which two NRT (*TraesCS6B02G044400* and *TraesCS6D02G035900*) showed a down-regulated expression in DS, but an up-regulated expression in DT, and two NR (*TraesCS6B02G024900* and *TraesCS7D02G073700*) specifically showed up-regulated expression in DT only (**Figure 4**).

### Identification of DELs

A total of 831 differentially expressed lncRNAs (DELs) were identified in DS under drought conditions compared to CK, including 446 up-regulated and 385 down-regulated DELs, respectively (**Supplementary Table S4**). A total of 788 DELs were identified in DT following drought treatment compared to CK, including 523 up-regulated and 265 down-regulated DELs, respectively (**Figures 2C, D**; **Supplementary Table S4**). The results showed that 104 DELs were commonly identified between DS and DT. In addition, 727 and 684 DELs were specific to DS and DT, respectively (**Figures 2C, D**). A total of 1515 DELs were identified in both varieties. Eight DELs were randomly selected for further qRT-PCR, including 5 up-regulated and 3 down-regulated DELs. The qRT-PCR and RNA-seq findings were highly consistent, indicating the reliability of the lncRNA data (**Supplementary Figure S7**).

### Identification of DEMs

A total of 155 differentially expressed miRNAs (DEMs) were identified in DS under drought conditions compared to CK, including 121 up-regulated and 34 down-regulated DEMs, respectively (**Supplementary Table S5**). A total of 123 DEMs were identified in DT following drought treatment compared to CK, including 81 up-regulated and 42 down-regulated DEMs, respectively (**Supplementary Table S5**). Venn diagram analysis showed that 69 DEMs were commonly identified between DS and DT. In addition, 86 and 54 DEMs were specific to DS and DT, respectively. A total of 209 DEMs were identified in both varieties (**Figures 2E, F**). To verify the accuracy of small RNA-Seq data, 6 up-regulated and 2 down-regulated DEMs were randomly selected for further qRT-PCR. The qRT-PCR and small RNA-seq findings were highly consistent, indicating the reliability of the miRNA data (**Supplementary Figure S7**).

### Screening of DEL-DEM-DEG regulatory modules in response to drought stress

Recent studies have shown are interactions among RNA molecules, such as lncRNA and miRNA, miRNA and mRNA, lncRNA and mRNA, and that these interactions form a regulatory network of lncRNA-miRNA-mRNA (Guttman and Rinn, 2012; Xu et al., 2020). By combining with miRNAs, lncRNAs reduce the ‘silencing effect’ of miRNAs on target genes, thereby regulating the target genes of miRNAs (Guttman and Rinn, 2012; Xu et al., 2020). To explore the lncRNA-miRNA-mRNA regulatory modules related to drought stress in wheat, DEMs were studied, and DEGs and DELs that had a targeting relationship with the DEMs but with the opposite expression pattern were searched. On this basis, DELs and DEGs having a targeting relationship with each other were retained. For example, by centering on up-regulated DEMs, we looked for DEGs and DELs that had a targeting relationship with these DEMs and with down-regulated expression. Or by centering on down-regulated DEMs, we looked for DEGs and DELs that had a targeting relationship with these DEMs and with up-regulated expression. Then, these DELs and DEGs having a targeting relationship with each other were then retained. Finally, the following two types of regulatory modules were obtained, namely DEL (down-regulated)-DEM (up-regulated)-DEG (down-regulated) and DEL (up-regulated)-DEM (down-regulated)-DEG (up-regulated).

**Figure 5:**
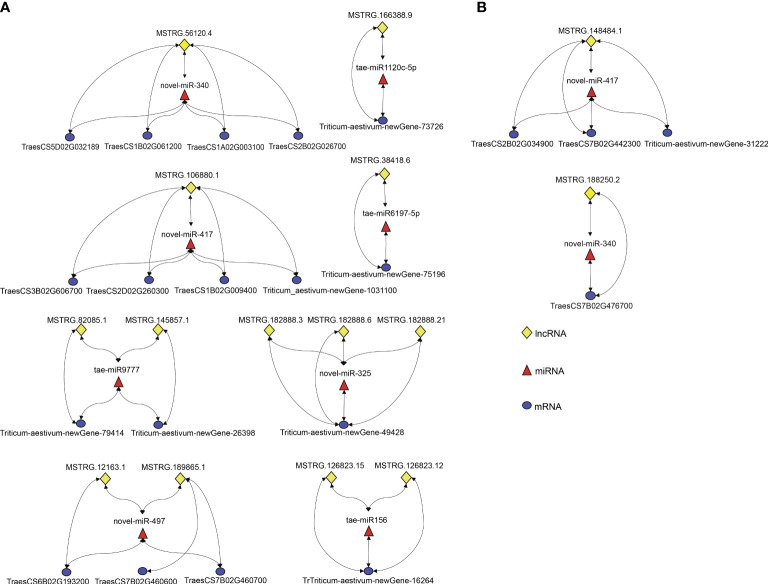
LncRNA-miRNA-mRNA regulatory modules associated with drought stress response were screened in DS **(A)** and DT **(B)**. Lines with arrows at both ends represent a targeting relationship between the two RNAs.

In the present study, 10 regulatory modules were identified in both varieties. All identified modules belonged to the first type, lncRNA (down-regulated)-miRNA(up-regulated)-mRNA (down-regulated) (**Figure 5**). A total of 8 DEL-DEM-DEG regulatory modules were identified in DS, including 9 up-regulated DEMs, 13 down-regulated DELs, and 17 down-regulated DEGs (**Figure 5**). Two DEL-DEM-DEG regulatory modules were identified in DT, including 2 up-regulated DEMs, 2 down-regulated DELs, and 4 down-regulated DEGs (**Figure 5**). DEMs novel-miR-417 and novel-miRNA-340 were commonly identified between DS and DT that targeted DELs and DEGs differently in the modules of the two wheat varieties. In DS, the targeted DEL of novel-miRNA-340 was MSTRG.56120.4 and the targeted DEGs were *TraesCS5D02G032189*, *TraesCS1B02G061200*, *TraesCS1A02G003100*, and *TraesCS2B02G026700*, respectively. But in DT, the targeted DEL and DEG of novel-miRNA-340 were MSTRG.188250.2 and *TraesCS7B02G476700*, respectively. Moreover, in DS, the targeted DEL and DEGs of novel-miRNA-417 were MSTRG.106880.1 and *TraesCS3B02G606700*, *TraesCS2D02G260300*, *TraesCS1B02G009400*, and *Triticum-aestivum-newGene-103110*, respectively. But in DT, the targeted DEL and DEGs of novel-miRNA-417 were MSTRG.148484.1 and *TraesCS2B02G034900*, *TraesCS7B02G442300*, and *Triticum-aestivum-newGene3-31222*, respectively (**Figure 5**). These results suggested that two wheat cultivars have different response mechanisms under drought stress.

**Figure 6:**
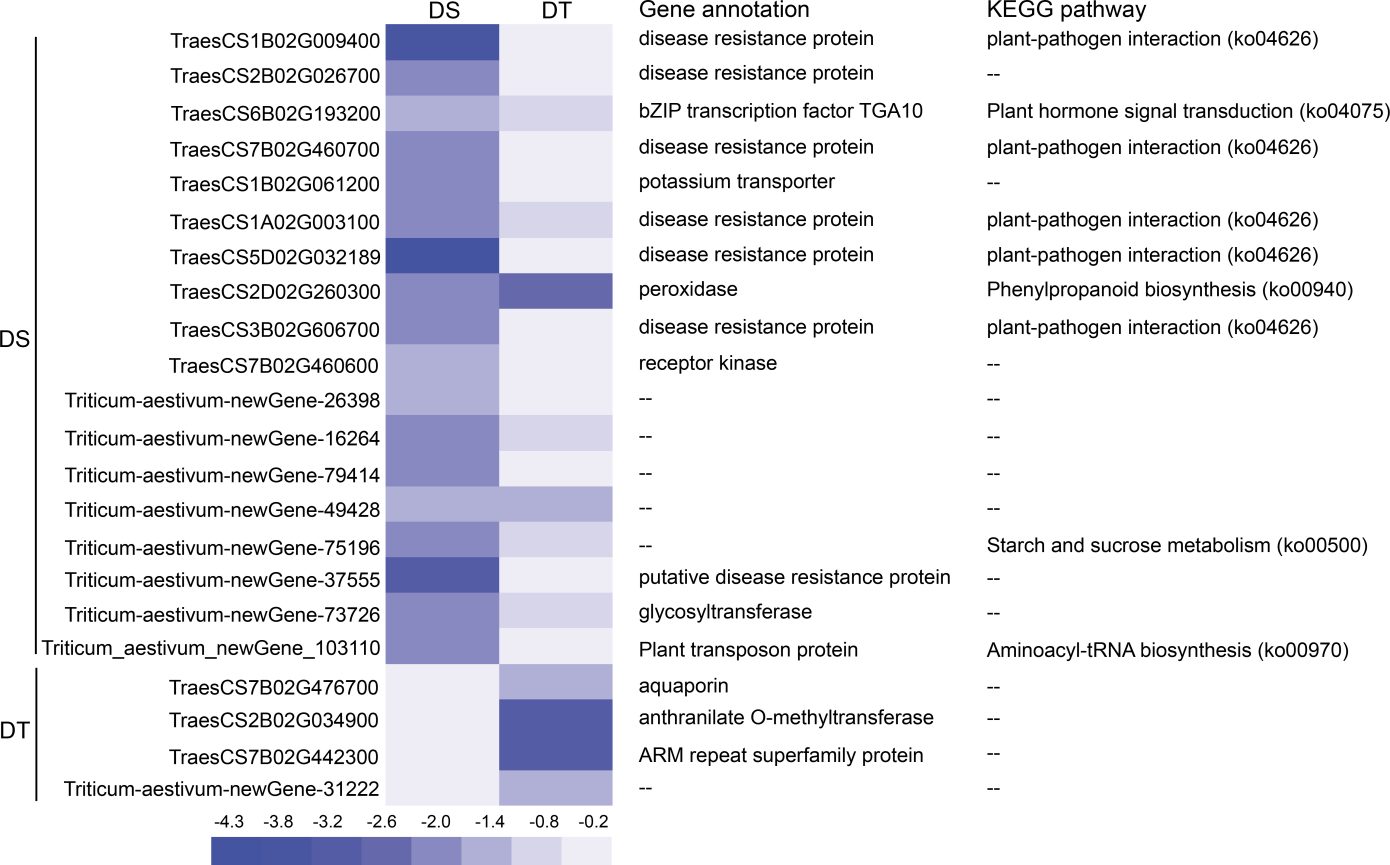
Heatmap of the expression of DEGs in lncRNA-miRNA-mRNA regulatory modules and their functional annotations. DS, drought-sensitive variety JM38, DT, drought-tolerant variety ZM13.

Gene annotation and KEGG pathways of 21 DEGs were analyzed in 10 modules. Among these genes, 16 were annotated to different functions, including six disease resistance proteins, one transcription factor, one potassium ion transporter, one peroxidase, one glycosyltransferase, one aquaporin, and one anthranilate O-methyltransferase. KEGG pathways involving these genes included plant-pathogen interaction pathway, plant hormone signal transduction pathway, phenylpropanoid biosynthesis pathway, starch and sucrose metabolism pathway, and aminoacyl-tRNA biosynthesis pathway (**Figure 6**).

### Expression patterns of candidate modules

**Figure 7:**
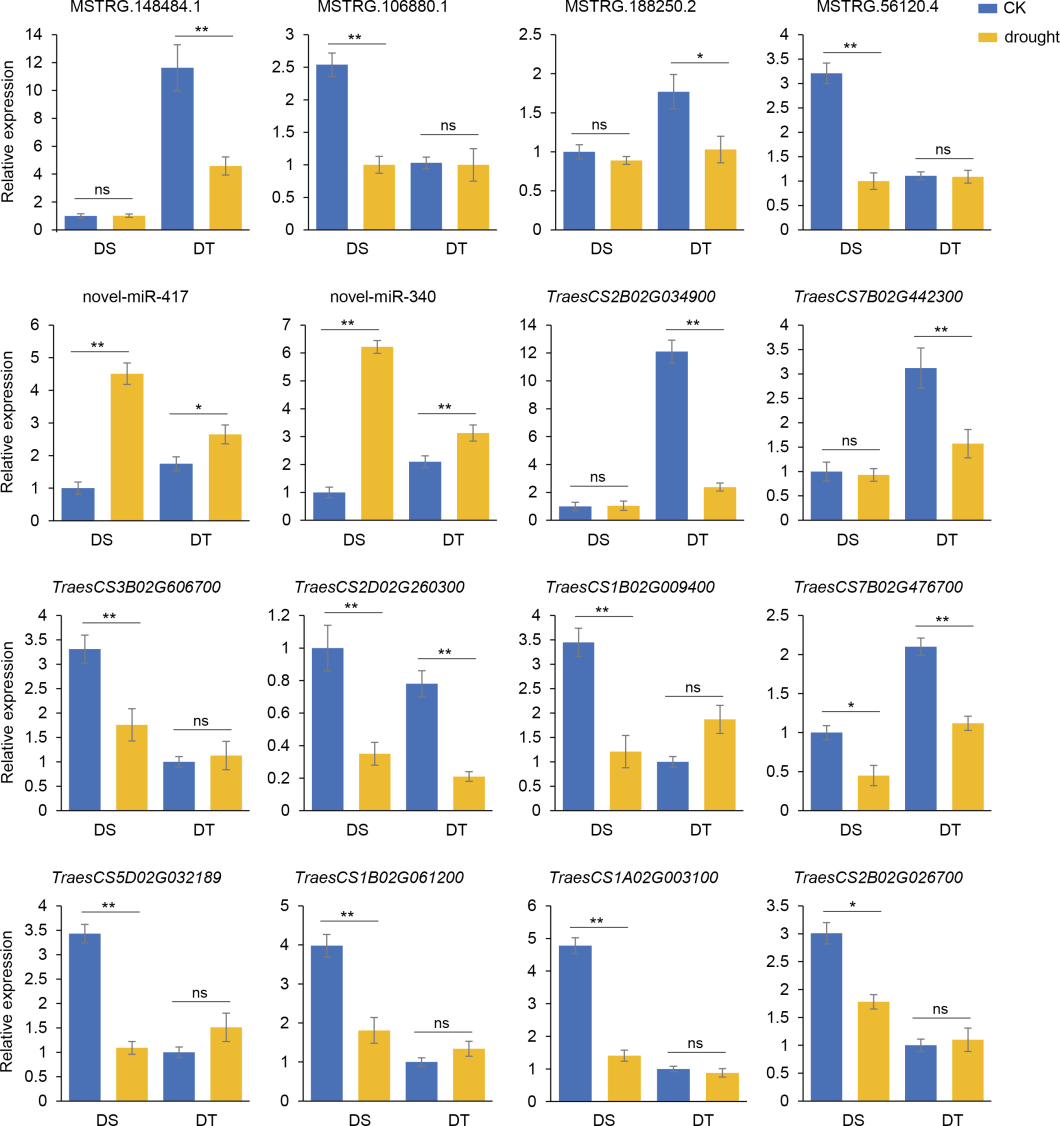
Validation of the expression patterns of lncRNA-miRNA-mRNA regulatory modules by qRT-PCR. The column represents the relative expression levels of qRT-PCR, and the broken line represents the log_2_(fold change) of RNA-seq or small RNA-seq. Values are means ± SD. *P < 0.05. **P < 0.01. ns, no significance.

Expression patterns of regulatory modules containing novel-miR-340 and novel-miR-417 were verified by qRT-PCR using the root cDNA of DS and DT as templates after four days of drought treatment. The results showed that the expression levels of MSTRG.148484.1 and MSTRG.188250.2 in DT were significantly decreased while the expression levels of MSTRG.106880.1 and MSTRG.56120.4 in DS were significantly decreased. Novel-miR-417 and novel-miR-340 were significantly upregulated in both cultivars after drought stress. *TraesCS2B02G034900* and *TraesCS7B02G442300* were significantly decreased only in DT after drought stress, while the expression levels of *TraesCS3B02G606700*, *TraesCS1B02G009400*, *TraesCS5D02G032189*, *TraesCS1B02G061200*, *TraesCS1A02G003100* and *TraesCS2B02G026700* in DS were significantly decreased. In addition, *TraesCS2D02G260300* and *TraesCS7B02G476700* were significantly downregulated after drought stress in both cultivars (**Figure 7**). These results indicated a competing expression pattern between the members of two candidate modules under drought stress conditions.

## Discussion

### Wheat improves drought tolerance by regulating the interaction of signaling transduction and nitrogen metabolism pathways

**Figure 8:**
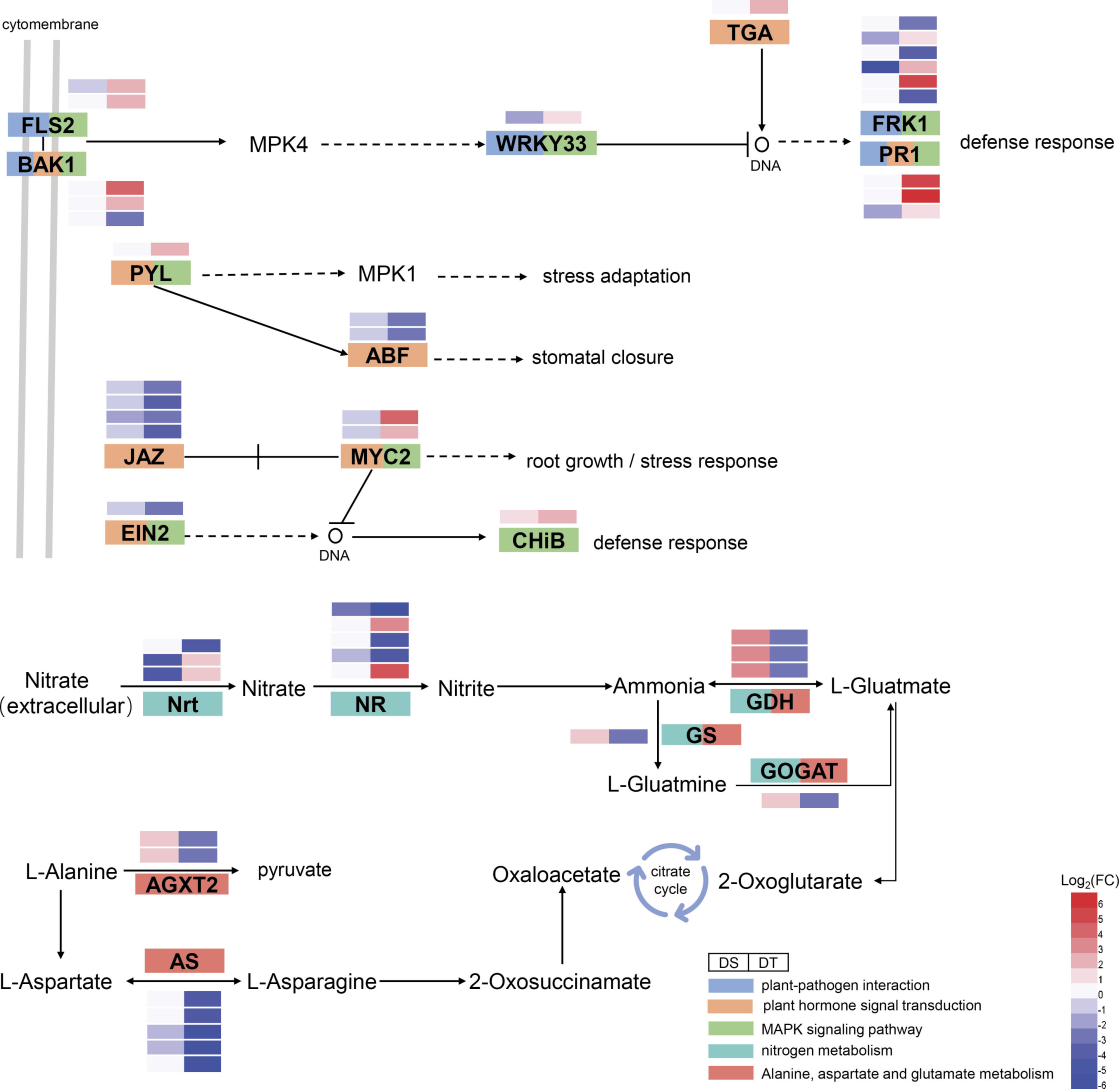
Expression patterns of DEGs in important pathways. Different colors represent different pathways. Genes containing more than two colors indicate their involvement in multiple pathways simultaneously. DS, drought-sensitive variety JM38, DT, drought-tolerant variety ZM13.

A large number of drought resistance-related DEGs were found enriched in three interconnected pathways, namely the plant-pathogen interaction pathway, plant hormone signaling transduction pathway, and MAPK signaling pathway (**Figure 8**). Some drought resistance-related DEGs were enriched in plant-pathogen interaction pathways, including threonine-protein kinase FLS2, brassinosteroid insensitive 1-associated receptor kinase 1 (BAK1), WRKY33, serine/threonine-protein kinase (FRK1), and pathogenesis-related protein PR1 which were also involved in the MAPK signal pathway (Figure 8). In addition, the ABA receptor protein PYL, transcription factor MYC2, and EIN2 in the MAPK signaling pathway were also involved in the plant hormone signal transduction pathway (**Figure 8**). Drought resistance-related DEGs which were enriched in phytohormone signal transduction pathways also included the ABF, transcription factor TGA, and JAZ. The above-mentioned drought resistance-related DEGs were also found to regulate stomatal opening and root growth through their interaction with each other, thereby enabling plants to adapt to stressful environments. For example, FLS2 and BAK1 can inhibit the expression of FRK1 and PR1 by indirectly affecting WRKY33, thus initiating the stress response mechanism in plants (**Figure 8**). PYL can not only improve plant adaptation to stress by activating mitogen-activated protein kinase (MPK1) but also indirectly affect plant stomatal closure by activating ABF (**Figure 8**). In addition, the interaction between JAZ and MYC2 can indirectly affect the growth of plant roots and improve their adaptability to stress. EIN2 can generate a defense response to the external environment by activating the expression of basic endochitinase B (CHiB) (**Figure 8**).

In addition to the above important pathways, a few drought resistance-related DEGs were significantly enriched in nitrogen metabolism and amino acid metabolism pathways with a close relationship observed between the two. In addition to being one of the nutrient elements necessary for plant growth and development, nitrogen also participates in and regulates physiological and biochemical processes, such as adaptation, repair, and compensation of plants under drought stress (Masclaux-Daubresse et al., 2010; Xu et al., 2012). Nitrate in the soil is transported to plant root cells by specific NRT, where it is reduced to nitrite by NR in the cytoplasm (Forde, 2002; Parker and Newstead, 2014). Nitrite is transferred to the chloroplast or plastid and reduced to ammonium nitrogen by nitrite reductase (NiR) (Stif et al., 2009). Ammonium nitrogen enters the GS/GOGAT cycle and is assimilated into organic nitrogen, such as glutamate, which is involved in the biosynthesis of other amino acids and proteins (Redinbaugh and Campbell, 1998). Studies have confirmed that external stress can induce the accumulation of nitrate in the roots of plants to improve plant resistance to external stress. Chen et al. (2012) showed that PEG-simulated drought promotes the accumulation of NO_3_^-^ in roots by altering the expression levels of *NRT1.5* to improve drought tolerance in plants. NR is known to mediate nitric oxide (NO) in plants (Pan et al., 2019). NR-induced NO improves tolerance to various stresses in plants (Kaya et al., 2020b). Kaya (2020a) showed the requirement of NR for salicylic acid-induced water stress tolerance in pepper plants. In this study, nitrogen uptake-related enzymes such as NRT and NR were significantly up-regulated in DT but significantly down-regulated in DS (**Figure 8**), suggesting that the nitrogen uptake was enhanced in drought-resistant wheat varieties. In addition, a few studies demonstrated that plants mainly accumulate proline by enhancing the proline biosynthesis pathway with glutamate as a prerequisite to maintain normal growth under osmotic stress conditions (Rhodes et al., 1987). In plants, glutamate mainly assimilates inorganic nitrogen through the GS/GOGAT cycle (Guan et al., 2014). Asparagine synthase (AS) can enhance plant disease and stress resistance by regulating nitrogen metabolism (Olea et al., 2004; Horst et al., 2010). In the present study, GDH, GS, and GOGAT were significantly down-regulated in DT and significantly up-regulated in DS (**Figure 8**). Further, five AS were significantly down-regulated in DT but not observed differently in DS (**Figure 8**). The above-mentioned drought-resistance-related DEGs may regulate the resistance of wheat to drought stress by participating in nitrogen metabolism and amino acid metabolic pathways.

### lncRNA-miRNA-mRNA regulatory modules in response to drought stress in wheat

To explore the lncRNA-miRNA-mRNA regulatory modules related to drought stress response in wheat, DEMs were studied, and DEGs and DELs that had a targeting relationship with DEMs and had the opposite expression pattern were searched. Finally, a total of 10 DEL-DEM-DEG regulatory modules were identified in both varieties (**Figure 5**). DELs, DEMs, and DEGs in each module were targeted at each other. Further, we used the gene expression profiling data published on the WheatOmics (Ma et al., 2021) data website (http://wheatomics.sdau.edu.cn/) to determine whether the regulatory modules screened in this study are tissue-specific. The expression analysis of DEGs from 10 modules in wheat roots, stems, leaves, spikes, and grains suggested that these genes were mainly expressed in wheat roots (**Supplementary Figure S8**). Therefore, we speculated that the regulatory modules screened in this study were tissue-specific and mainly regulated the expression of genes in wheat roots under drought stress. Using the published miRNA data (Akdogan et al., 2016) of wheat after drought stress, we verified the expression of 4 known miRNAs in the regulatory modules screened in this study from which 2 of the miRNAs (tae-miR156 and tae-miR1120c-5p) has been reported an up-regulated expression pattern previously. In addition, we also used the degradome data on the miRNA target gene prediction platform WPMIAS (Whole-degradome-based Plant MicroRNA-Target Interaction Analysis Server) (Fei et al., 2019) to verify the prediction results of tae-miR156 target genes. The results showed that most of the predicted tae-miR156 target genes (85.71%) based on the degradome were also predicted in this study.

**Figure 9:**
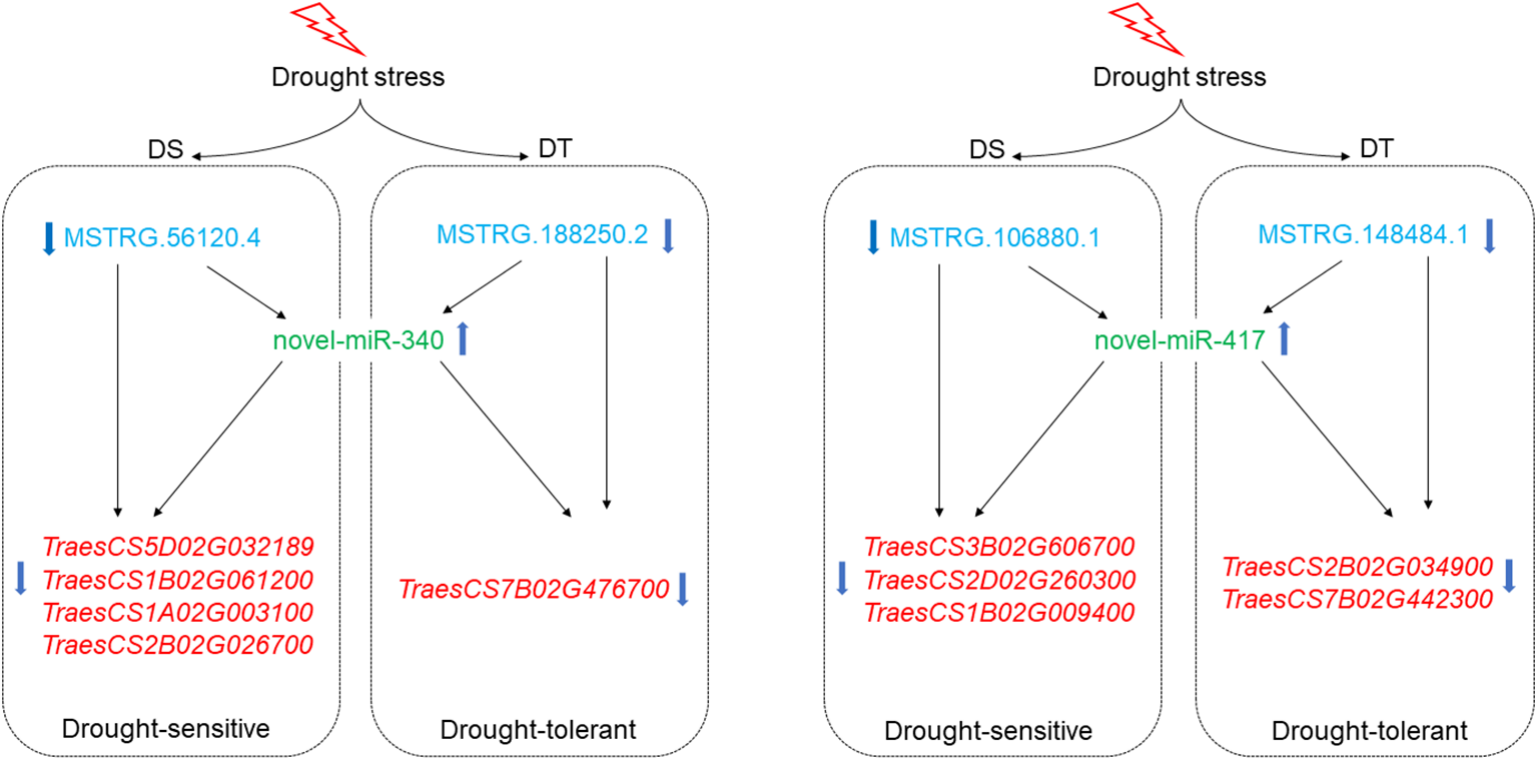
Prediction of the regulatory patterns of two candidate lncRNA-miRNA-mRNA modules in the two wheat cultivars.

Among these modules, DEMs novel-miR-340 and novel-miR-417 were commonly identified between DS and DT which targeted DELs and DEGs differently in the modules of the two varieties (**Figure 5**). Combined with qRT-PCR results, it was speculated that in DS, MSTRG.56120.4 can inhibit *TraesCS5D02G032189*, *TraesCS1B02G061200*, *TraesCS1A02G003100*, and *TraesCS2B02G026700* expression by activating novel-miRNA-340 (**Figure 9**). In DT, novel-miRNA-340 was activated by MSTRG.188250.2, resulting in the down regulation of *TraesCS7B02G476700* (**Figure 9**). The annotation function of *TraesCS7B02G476700* is aquaporin (AQP). Plant AQP can not only promote long-distance water transport from plant roots to leaves but also intracellular and extracellular transmembrane transport of water as a stress response mechanism (Maurel et al., 2009). By analyzing the published gene expression profiling data (http://wheatomics.sdau.edu.cn/), we found that the expression level of *TraesCS7B02G476700* was also decreased when wheat subjected to low phosphorus (Oono et al., 2013) and salt stress (Zhang et al., 2016) (**Supplementary Figure S8**). In addition, the ortholog of *TraesCS7B02G476700* gene in rice, *rMip1*, was also induced by water and salt stress (Liu et al., 1994). The qRT-PCR results also confirmed that novel-miR-417 and its target-related DELs and DEGs showed an expression trend consistent with the sequencing results of both varieties. Among them, DEG *TraesCS2D02G260300* targeted by novel-miR-417 in DS is a peroxidase (POD) gene. POD can regulate the balance of reactive oxygen species in organisms by catalyzing the redox of H_2_O_2_, and play an important role in plant growth and development and abiotic stress response (Meloni et al., 2003). By analyzing the expression profile data of *TraesCS2D02G260300* under different abiotic stresses, we found that the transcription of *TraesCS2D02G260300* can be induced by a variety of abiotic stresses. For example, the expression level of *TraesCS2D02G260300* was decreased under low phosphorus (Oono et al., 2013), heat (Kumar et al., 2015), and salt stress (Zhang et al., 2016), whereas increased under cold stress (Li et al., 2015) (**Supplementary Figure S8**). In summary, these two regulatory modules centered on novel-miR-340 and novel-miR-417 may play a role in wheat drought stress response, and regulate different genes in the two drought-resistant types of wheat, thus showing different drought resistance performance.

## Data availability statement

The original contributions presented in the study are publicly available. This data can be found here: NCBI, PRJNA838787 and PRJNA837867.

## Author contributions

NL: conceptualization, experimentation, writing of the original draft, reviewing, and editing. TL: conceptualization, experimentation, reviewing, and editing. FG: experimentation and data curation. JY: data curation. YS: experimentation. SW: data curation and editing. DS: conceptualization, writing, reviewing, and editing. All authors contributed to the article and approved the submitted version.

## Funding

This work was funded by Fundamental Research Program of Shanxi Province (20210302124148), Scientific and Technologial Innovation Programs of Higher Education Institutions in Shanxi (2021L124) and the Scientific and Technological Innovation Programs of Shanxi Agricultural University (2020BQ30).

## Conflict of interest

The authors declare that the research was conducted in the absence of any commercial or financial relationships that could be construed as a potential conflict of interest.

## Publisher’s note

All claims expressed in this article are solely those of the authors and do not necessarily represent those of their affiliated organizations, or those of the publisher, the editors and the reviewers. Any product that may be evaluated in this article, or claim that may be made by its manufacturer, is not guaranteed or endorsed by the publisher.

## Supplementary material

Supplementary Figure 1 Number of predicted lncRNAs using the four analytical methods. CPC, coding potential calculator, CNCI, coding-non-coding index, Pfam, protein family, CPAT, coding potential assessment tool.

Supplementary Figure 2 Statistical plot of lncRNAs.

Supplementary Figure 3 Length distribution of miRNAs.

Supplementary Figure 4 Validation of the expression patterns of randomly selected DEGs by qRT-PCR. The column represents the relative expression level of qRT-PCR, and the broken line represents the log_2_(fold change) of RNA-Seq. Positive values on the y axis represent up-regulation of genes, and negative values represent down-regulation of genes.

Supplementary Figure 5 GO enrichment analysis of drought resistance-related DEGs.

Supplementary Figure 6 KEGG enrichment analysis of drought resistance-related DEGs. **(A)** Scatter plot of KEGG pathway enrichment analysis. Each circle in the figure represents a KEGG pathway, the ordinate represents the name of the pathway, and the abscissa represents the enrichment factor. The larger the enrichment factor, the more significant the enrichment level of DEGs in the pathway. **(B)** Histogram of KEGG enrichment analysis. Only KEGG pathways with enriched DEGs greater than or equal to 4 are listed. The numbers in the figure represent the number and percentage of DEGs enriched in each KEGG pathway.

Supplementary Figure 7 Validation of the expression patterns of randomly selected differentially expressed lncRNAs and differentially expressed miRNAs by qRT-PCR. The column represents the relative expression levels of qRT-PCR, and the broken line the log2(fold change) of RNA-seq or small RNA-seq.

Supplementary Figure 8 Expression of important DEGs in the lncRNA-miRNA-mRNA regulatory module in different tissues of wheat and under various abiotic stresses. **(A)** Expression of DEGs in different tissues of wheat. **(B)** Expression of important DEGs under low phosphorus stress. **(C)** Expression of important DEGs under cold stress. **(D)** Expression of important DEGs under heat stress. **(E)** Expression of important DEGs under salt stress.
