# Supplementary figures and images for: Identification of long non-coding RNA-microRNA-mRNA regulatory modules and their potential roles in drought stress response in wheat (Triticum aestivum L.)
Source: Front Plant Sci. 2022 Oct 11;13:1011064. doi: 10.3389/fpls.2022.1011064 (PMC9592863; doi:10.3389/fpls.2022.1011064)

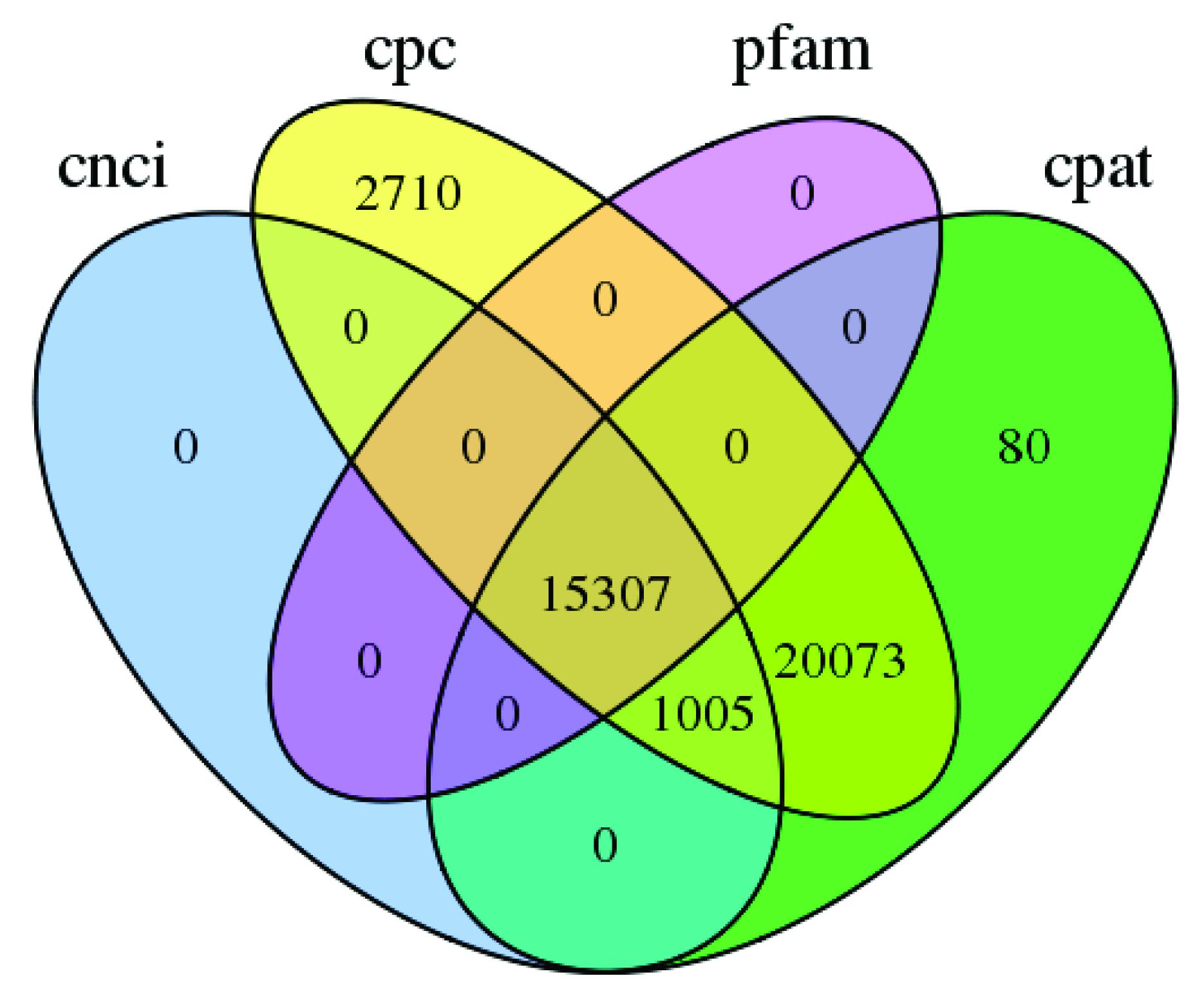

Supplement: Supplementary Figure 1 — Number of predicted lncRNAs using the four analytical methods. CPC, coding potential calculator, CNCI, coding-non-coding index, Pfam, protein family, CPAT, coding potential assessment tool. [file Image_1.tif]

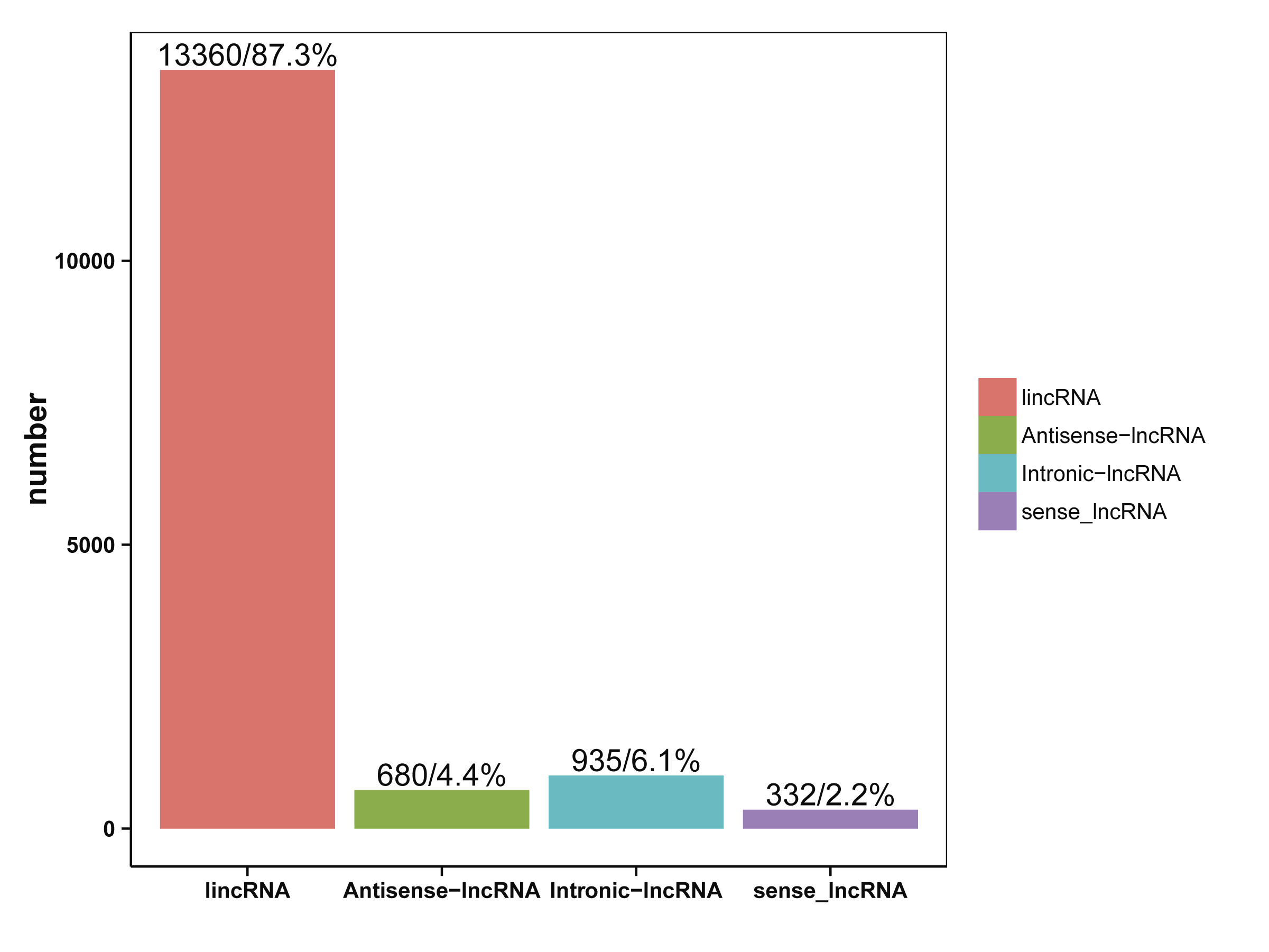

Supplement: Supplementary Figure 2 — Statistical plot of lncRNAs. [file Image_2.tif]

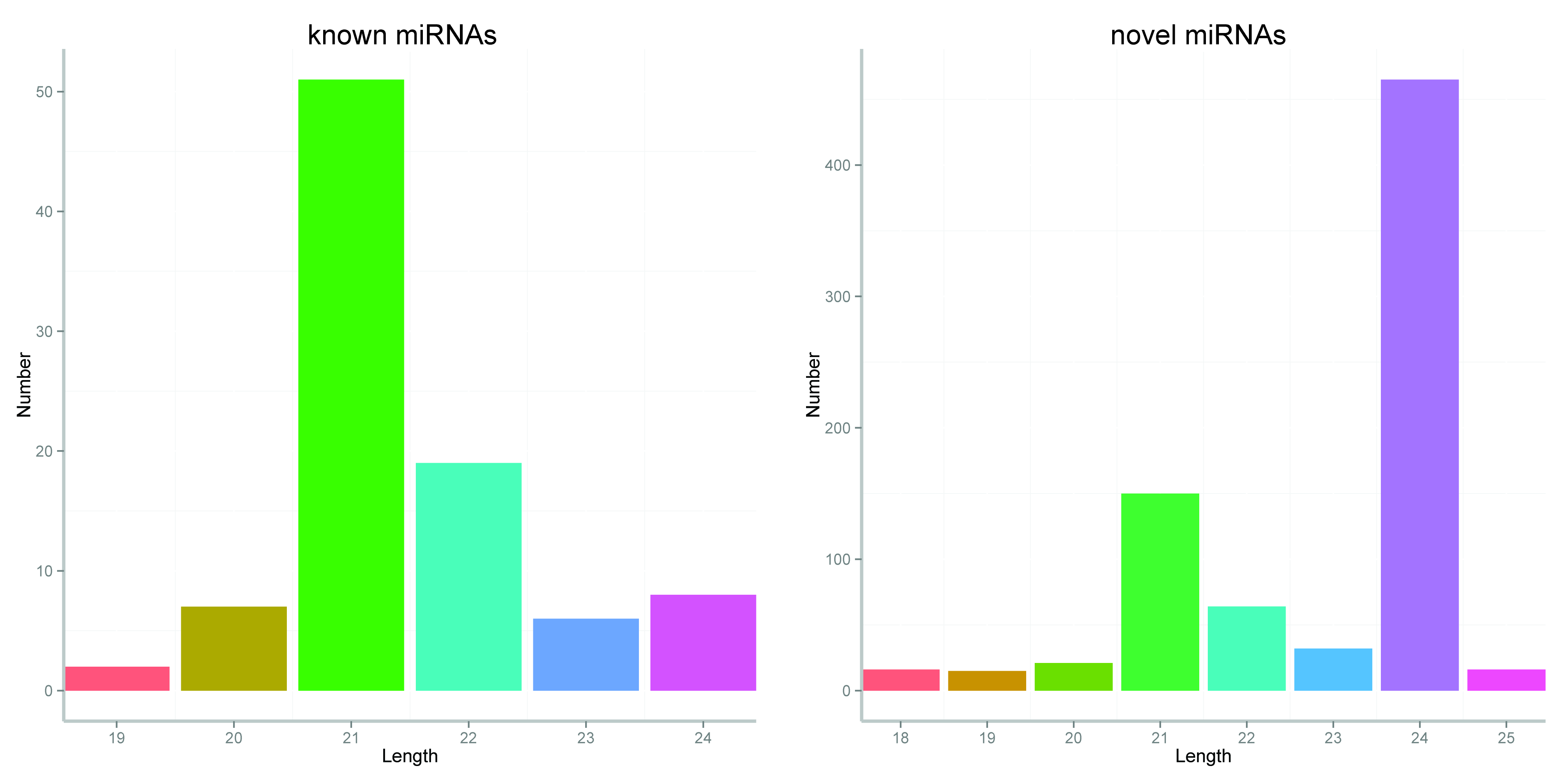

Supplement: Supplementary Figure 3 — Length distribution of miRNAs. [file Image_3.tif]

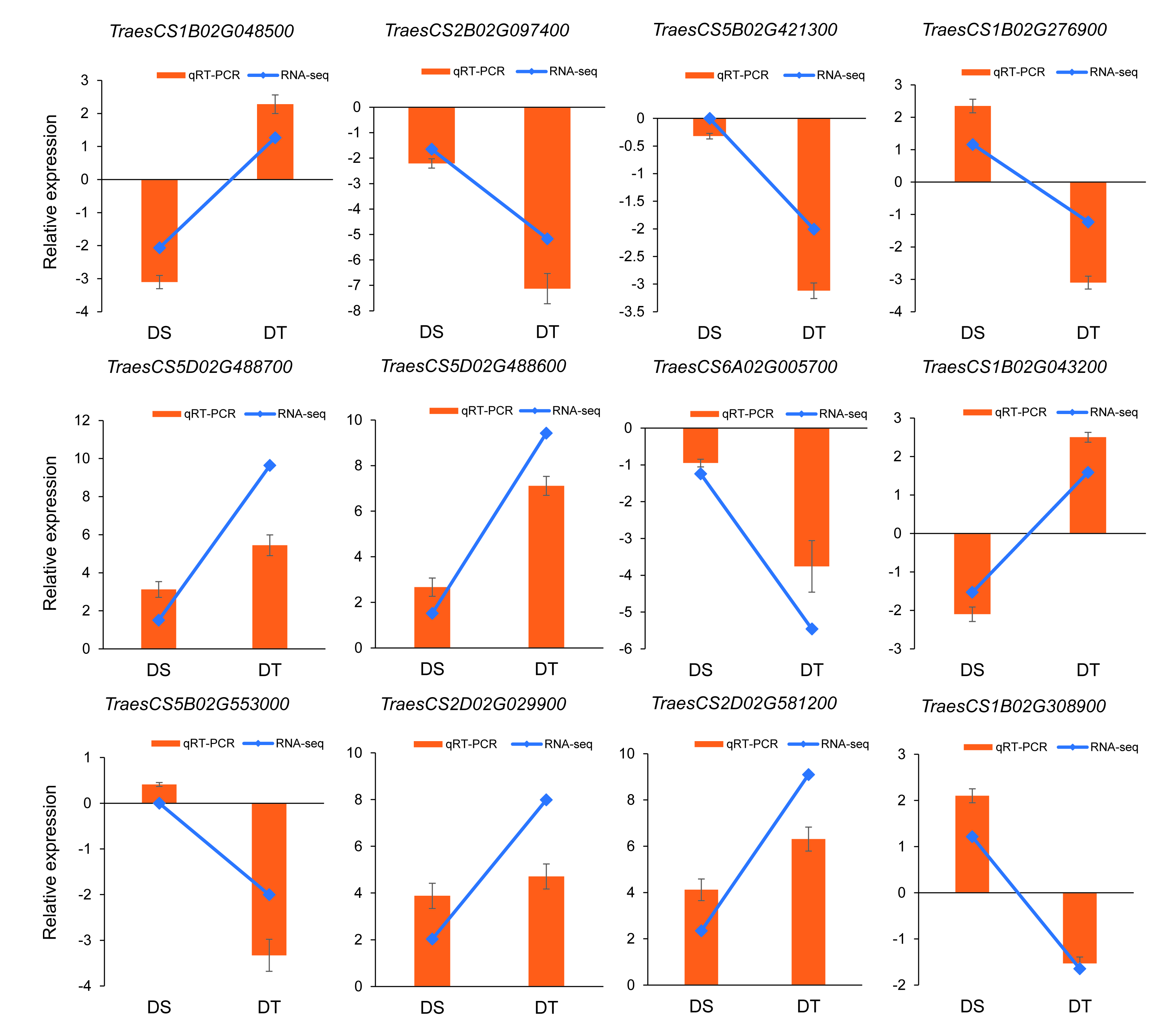

Supplement: Supplementary Figure 4 — Validation of the expression patterns of randomly selected DEGs by qRT-PCR. The column represents the relative expression level of qRT-PCR, and the broken line represents the log2(fold change) of RNA-Seq. Positive values on the y axis represent up-regulation of genes, and negative values represent down-regulation of genes. [file Image_4.tif]

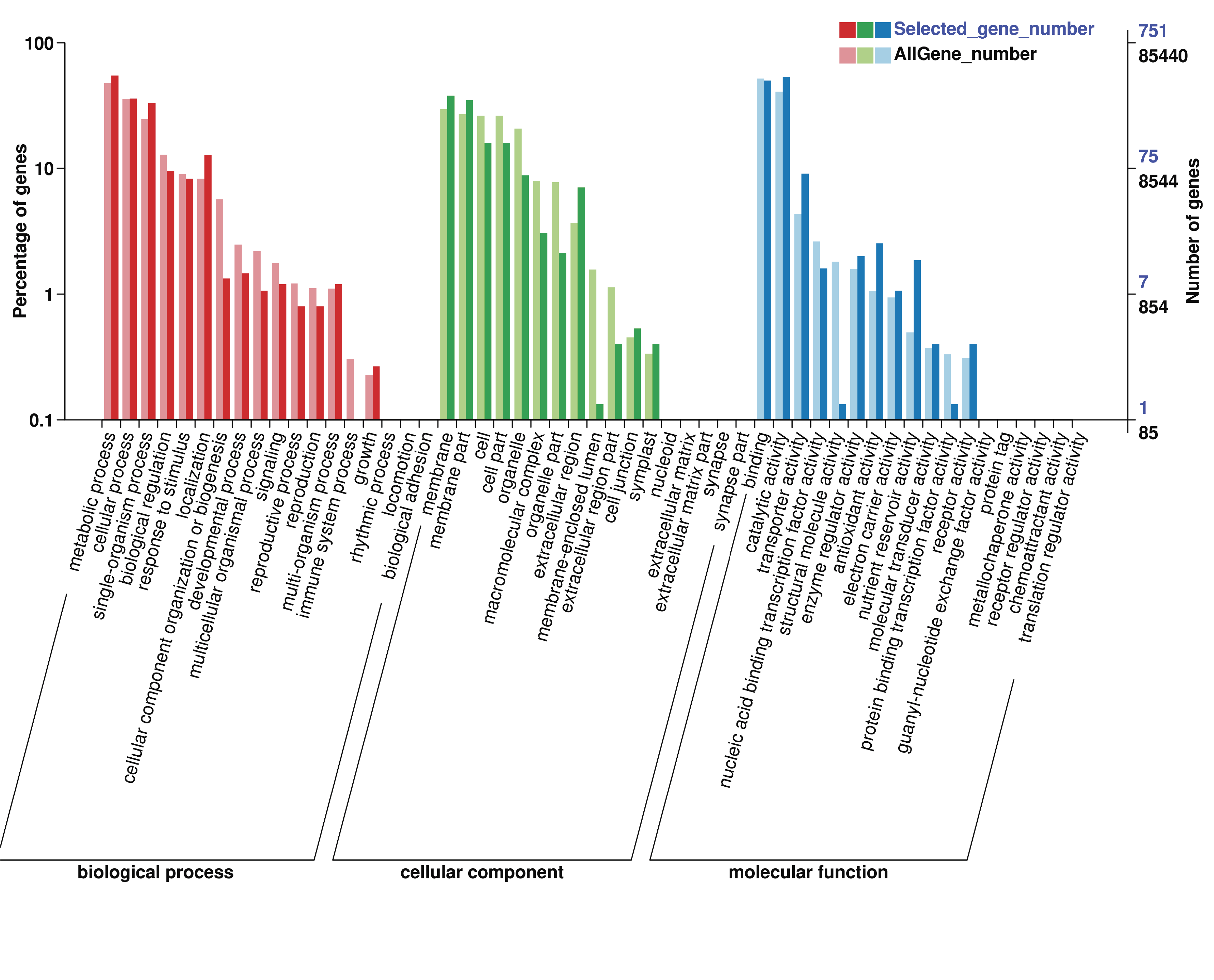

Supplement: Supplementary Figure 5 — GO enrichment analysis of drought resistance-related DEGs. [file Image_5.tif]

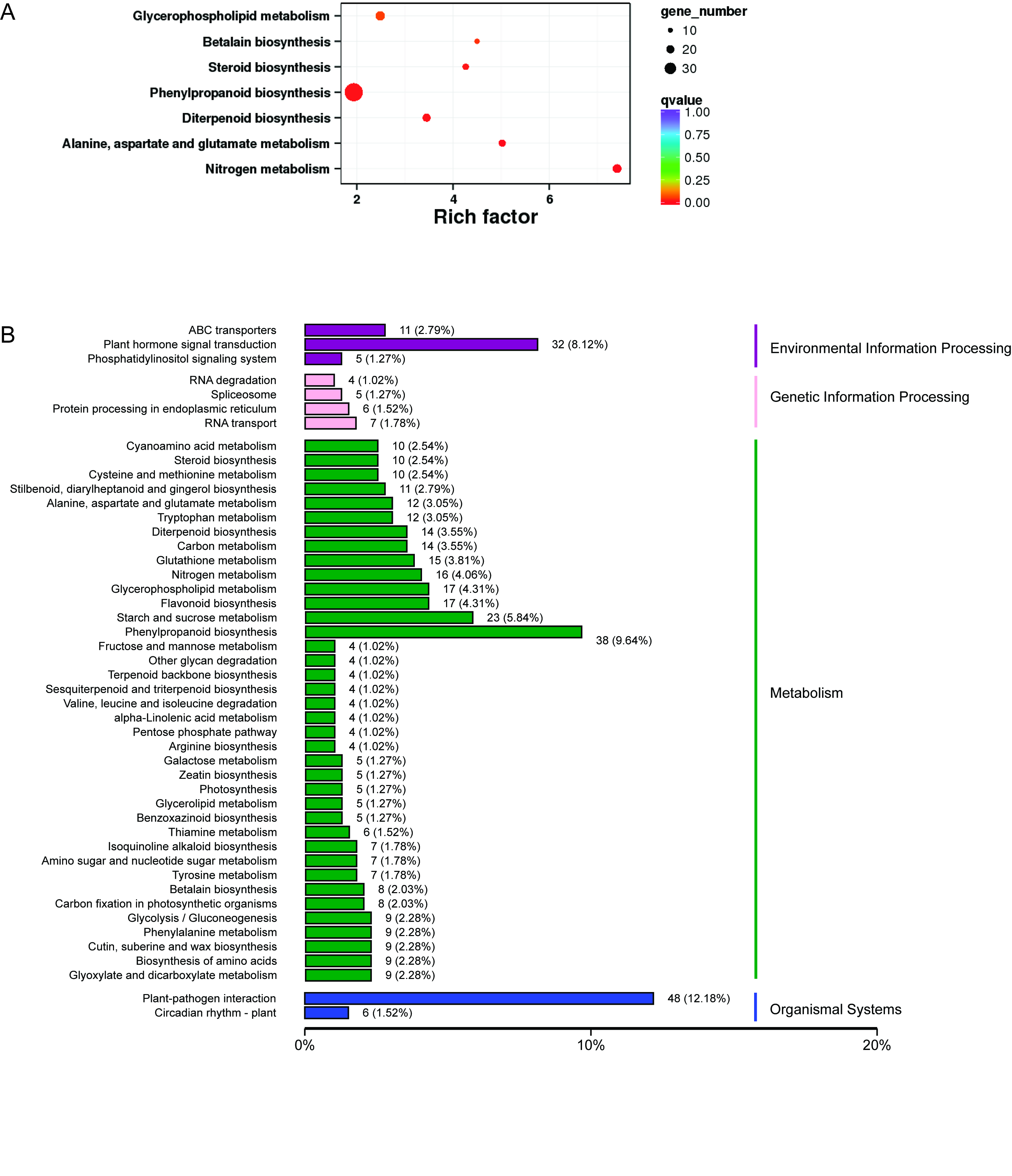

Supplement: Supplementary Figure 6 — KEGG enrichment analysis of drought resistance-related DEGs. (A) Scatter plot of KEGG pathway enrichment analysis. Each circle in the figure represents a KEGG pathway, the ordinate represents the name of the pathway, and the abscissa represents the enrichment factor. The larger the enrichment factor, the more significant the enrichment level of DEGs in the pathway. (B) Histogram of KEGG enrichment analysis. Only KEGG pathways with enriched DEGs greater than or equal to 4 are listed. The numbers in the figure represent the number and percentage of DEGs enriched in each KEGG pathway. [file Image_6.tif]

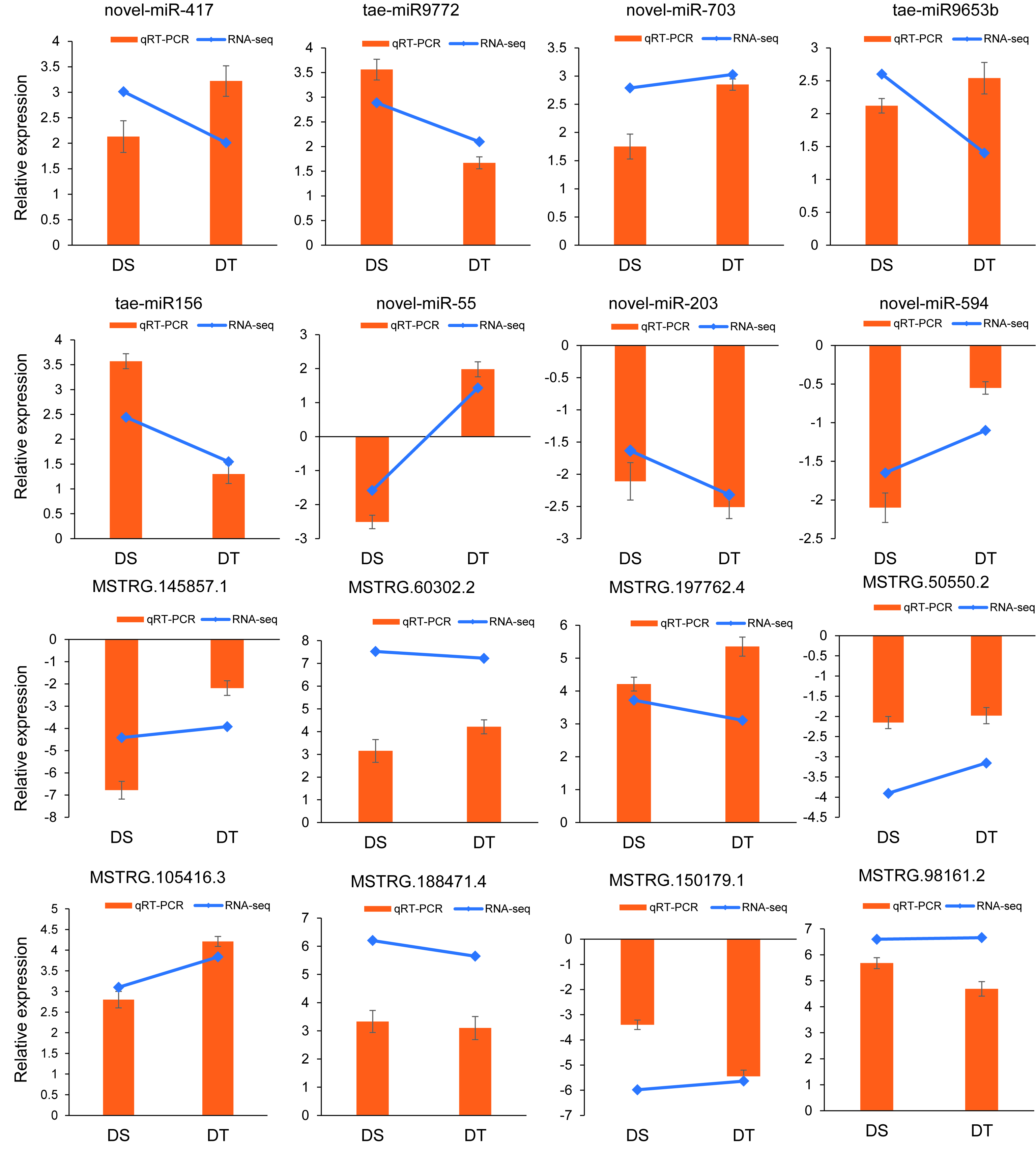

Supplement: Supplementary Figure 7 — Validation of the expression patterns of randomly selected differentially expressed lncRNAs and differentially expressed miRNAs by qRT-PCR. The column represents the relative expression levels of qRT-PCR, and the broken line the log2(fold change) of RNA-seq or small RNA-seq. [file Image_7.tif]

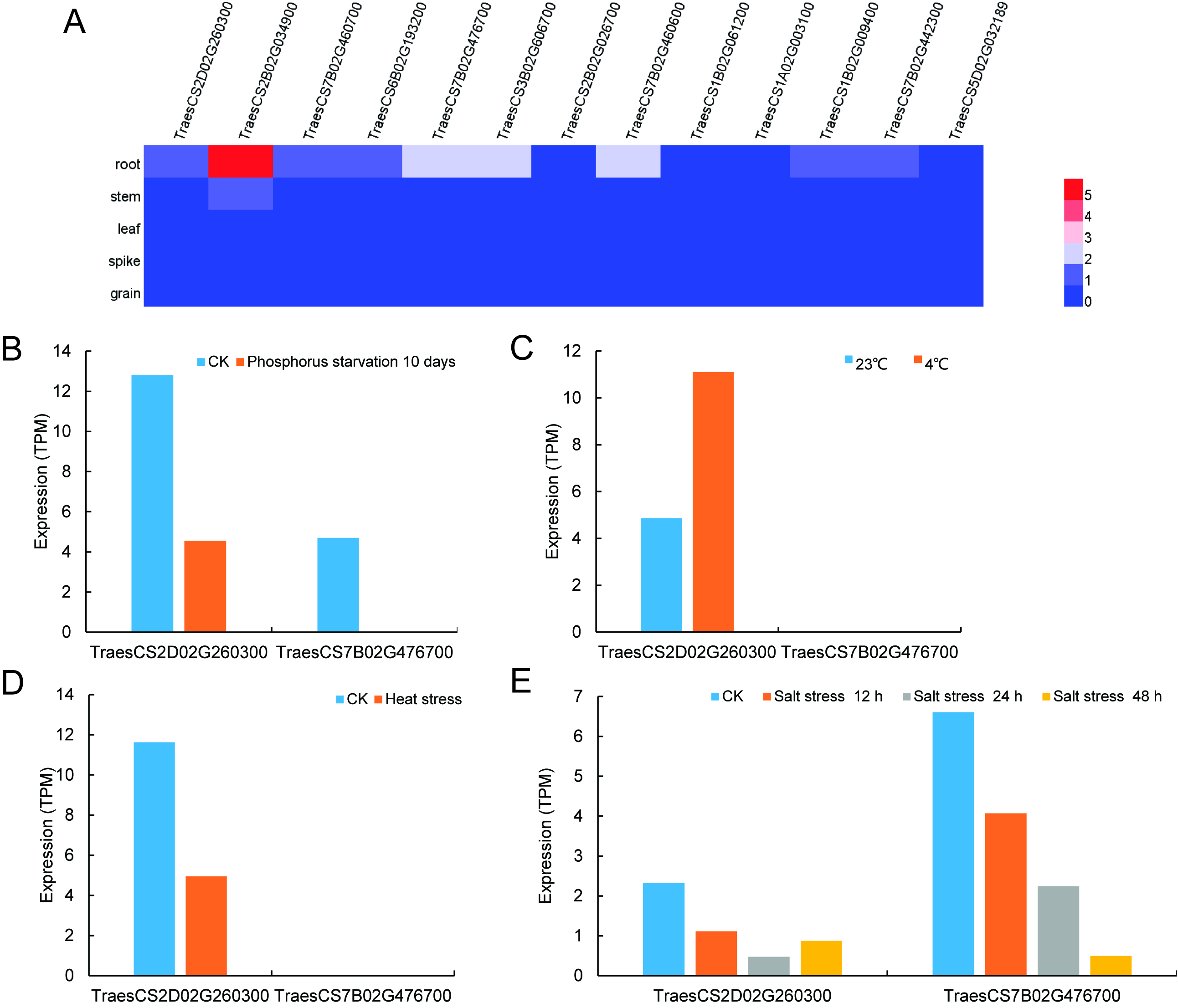

Supplement: Supplementary Figure 8 — Expression of important DEGs in the lncRNA-miRNA-mRNA regulatory module in different tissues of wheat and under various abiotic stresses. (A) Expression of DEGs in different tissues of wheat. (B) Expression of important DEGs under low phosphorus stress. (C) Expression of important DEGs under cold stress. (D) Expression of important DEGs under heat stress. (E) Expression of important DEGs under salt stress. [file Image_8.tif]
