# Supplementary material for: Identification of long non-coding RNA-microRNA-mRNA regulatory modules and their potential roles in drought stress response in wheat (Triticum aestivum L.)
Source: Front Plant Sci. 2022 Oct 11;13:1011064. doi: 10.3389/fpls.2022.1011064 (PMC9592863; doi:10.3389/fpls.2022.1011064)
Supplement: Supplementary file 9 [file Table_1.docx]

| Supplementary Table S1 Primers for qRT-PCR | |
| --- | --- |
| Primer name^a^ | Sequence (5' to 3') |
| TraesCS6A02G005700-F | GCAGTGCCCGTAGAGAGTG |
| TraesCS6A02G005700-R | ACATGGCCCTTGTCGAAGTC |
| TraesCS2B02G097400-F | CACCCCAAACAGTCTCTCCA |
| TraesCS2B02G097400-R | CTGCCTCCTTACGGTTCACC |
| TraesCS1B02G043200-F | TCCTGGCGGTGGAGTTGAT |
| TraesCS1B02G043200-R | CGTCCCAATGCCCTGCAAT |
| TraesCS1B02G048500-F | GCATGGCTTCTTCGAGTTGC |
| TraesCS1B02G048500-R | ACGCCGAGAATGTTAGCCTC |
| TraesCS1B02G276900-F | GAGACGAGCCCCAAGAAGAC |
| TraesCS1B02G276900-R | GTACTTCTTACAGGGCGGGC |
| TraesCS1B02G308900-F | GTGGTAACCCTGTCAGCACA |
| TraesCS1B02G308900-R | AGCCAACAACGAACGCATTC |
| TraesCS5D02G488700-F | CCCATGTGACACCCAGCTAG |
| TraesCS5D02G488700-R | CTTTCAACAAGCACGTCGGG |
| TraesCS5D02G488600-F | TGTGCTCTTCATCCTCGACG |
| TraesCS5D02G488600-R | AACCAAAGAGTACGCCCCAG |
| TraesCS5B02G553000-F | CTGGTCCTAGGGGTGCTTC |
| TraesCS5B02G553000-F | TTGAACACTAGGACGTCGCC |
| TraesCS5B02G421300-F | CATGTTGGCCGTGCTCATG |
| TraesCS5B02G421300-R | TGTCAGTTGGCACGGCAG |
| TraesCS2D02G029900-F | GCTTGACTTCCCGATCGACA |
| TraesCS2D02G029900-R | TGTGGGCTCTTGTTGTCGAA |
| TraesCS2D02G581200-F | CTCCCAACTCAAGGCCAGG |
| TraesCS2D02G581200-R | GCCTTCTCATTAGCCTCCGG |
| TraesCS2B02G034900-F | GAGAATAGCTACGCCGCCAA |
| TraesCS2B02G034900-R | ACGATGAAGAGAGCGTTCGG |
| TraesCS7B02G442300-F | ATCTCCACAACTGCGCTGAA |
| TraesCS7B02G442300-R | AATCTCTTGCCGCAGTGTCA |
| TraesCS3B02G606700-F | TGAACGAGCTGGGAGGGATA |
| TraesCS3B02G606700-R | ATGTCTCGTTCTGCCCCAAG |
| TraesCS2D02G260300-F | TGCCCATGGACACTGTGAC |
| TraesCS2D02G260300-R | GAGCTCAGTAGGCCCATTCC |
| TraesCS1B02G009400-F | AGGAGCAAGGAAGATGGCAC |
| TraesCS1B02G009400-R | TTCCGTCAGCTTCCCAATCC |
| TraesCS7B02G476700-F | AGCATCCATGAACCCTGCAA |
| TraesCS7B02G476700-R | CCGCTAATAGGCCCAACGAT |
| TraesCS5D02G032189-F | AAGCCGGGTTCTCAGTTCTG |
| TraesCS5D02G032189-R | GGTGGCGTTGAAGACTAGCT |
| TraesCS1B02G061200-F | GCTTAGCATCACAGGGACCG |
| TraesCS1B02G061200-R | TGCCCAGTGTACCCAAGAAG |
| TraesCS1A02G003100-F | AGGCTGTGGTCATGACGAAG |
| TraesCS1A02G003100-R | CATGTGCACTCCTCCCTTGT |
| TraesCS2B02G026700-F | AACCATGCCAAATCCGAGGA |
| TraesCS2B02G026700-F | GCCTCCTCAATGACGATGCT |
| MSTRG.50550.2-F | TGTGTGAGGCCGCTGTATTT |
| MSTRG.50550.2-R | CGAGGAACAATGTGGACCCA |
| MSTRG.145857.1-F | CTCTCCAACCTGTCCGTCTG |
| MSTRG.145857.1-R | ATCTGGTTGCGACAAGGTGT |
| MSTRG.60302.2-F | TCTCTCAACTCGCCGAACAC |
| MSTRG.60302.2-R | CCGGGCCACTTCTTCTCTTT |
| MSTRG.197762.4-F | GTGAGACCAAGTGCCTAGACA |
| MSTRG.197762.4-R | GGCCCACAGTGAAATTCCTA |
| MSTRG.105416.3-F | GGGCAGCCTGATAAGGAAGA |
| MSTRG.105416.3-R | GCCTTCAGTCTCTGCGTACA |
| MSTRG.188471.4-F | CTTCGCACACACTCCTTTGC |
| MSTRG.188471.4-R | TGGATCATGGTGAGTCGTGC |
| MSTRG.150179.1-F | CACCGCGCCTAAGTGTTTTA |
| MSTRG.150179.1-R | AAGGGCGACAGTTCTAGCAC |
| MSTRG.98161.2-F | CCACTACCCCTGACACAACC |
| MSTRG.98161.2-R | AGTCGAAGCAGTGATGGTGG |
| MSTRG.148484.1-F | GGCCTACCCGTACCTAAGAC |
| MSTRG.148484.1-R | AGCAACACCTCCCGAATGTG |
| MSTRG.106880.1-F | CATCAAGCTGCCCTTTGACG |
| MSTRG.106880.1-R | ACATCGTCGTTCCTCCAACC |
| MSTRG.188250.2-F | GGGTCATCTTCTCACCGGAC |
| MSTRG.188250.2-R | ACTCTGTGGCTGCCTGATTC |
| MSTRG.56120.4-F | TGCTGCTGAATGTGTTGTGC |
| MSTRG.56120.4-R | CCACGCACCAAAAAGACCAG |
| Novel-miR-417-F | CCTGGTGGTCATGGCGAG |
| tae-miR9772-F | CCCTGAGATGAGATTACCCCATAC |
| Novel-miR-703-F | GGCCTCTCAGGCCCTGA |
| tae-miR9653b-F | TGGCCAAGGTCTCTTGAGG |
| tae-miR156-F | ACTGACAGAAGAGAGTGAGCACA |
| Novel-miR-55-F | GGAGAATCTGGCAGAAGAACTGG |
| Novel-miR-203-F | TTTGCATCTTGTCCGGTCTCTGACA |
| Novel-miR-594-F | GTCCGATCTGGGCTGTTGG |
| Novel-miR-340-F | CGCAGTGAGATGATGAATG |
| TaEF-1a-F | TGGTGTCATCAAGCCTGGTATGGT |
| TaEF-1a-R | ACTCATGGTGCATCTCAACGGACT |
| TaU6-F | GGGGACATCCGATAAAATTGG |
| TaU6-R | GGACCATTTCTCGATTTGTGC |

^a^ F, forward primer; R, reverse primer
