# Supplementary material for: Identification of long non-coding RNA-microRNA-mRNA regulatory modules and their potential roles in drought stress response in wheat (Triticum aestivum L.)
Source: Front Plant Sci. 2022 Oct 11;13:1011064. doi: 10.3389/fpls.2022.1011064 (PMC9592863; doi:10.3389/fpls.2022.1011064)
Supplement: Supplementary file 10 [file Table_2.docx]

Supplementary Table S2 Statistical results of miRNAs in each sample

| sample ID ^a^ | Known-miRNAs | Novel-miRNAs | Total |
| --- | --- | --- | --- |
| DSCK1 | 80 | 761 | 841 |
| DSCK2 | 81 | 758 | 839 |
| DSCK3 | 78 | 755 | 833 |
| DST1 | 73 | 685 | 758 |
| DST2 | 78 | 744 | 822 |
| DST3 | 67 | 690 | 757 |
| DTCK1 | 76 | 747 | 823 |
| DTCK2 | 79 | 736 | 815 |
| DTCK3 | 80 | 732 | 812 |
| DTT1 | 81 | 686 | 767 |
| DTT2 | 73 | 682 | 755 |
| DTT3 | 75 | 689 | 764 |
| Total | 93 | 779 | 872 |

^a^ DSCK1-3, samples of drought-sensitive wheat varieties under control conditions; DST1-3, samples of drought-sensitive wheat varieties under drought conditions; DTCK1-3 samples of drought-tolerant wheat varieties under control conditions; DTT1-3, samples of drought-tolerant wheat varieties under drought conditions.
