# Supplementary material for: Identification of long non-coding RNA-microRNA-mRNA regulatory modules and their potential roles in drought stress response in wheat (Triticum aestivum L.)
Source: Front Plant Sci. 2022 Oct 11;13:1011064. doi: 10.3389/fpls.2022.1011064 (PMC9592863; doi:10.3389/fpls.2022.1011064)
Supplement: Supplementary file 14 [file Table_6.docx]

**Supplementary Table S6 Comparison of tae-miR156 target gene prediction results**

| miRNA | Prediction of tae-miR156 target genes in this study | Prediction of tae-miR156 target genes using WPMIAS |
| --- | --- | --- |
| tae-miR156 | *TraesCS1A02G071800, TraesCS1A02G190900, TraesCS1A02G436900, TraesCS1B02G080700, TraesCS1A02G107500, TraesCS1B02G373500, TraesCS1D02G310400, TraesCS2A02G103300, TraesCS2A02G232400, TraesCS2A02G277500, TraesCS2A02G350100, TraesCS2A02G350300, TraesCS2A02G360300, TraesCS2A02G413900, TraesCS2A02G429800, TraesCS2A02G518300, TraesCS2B02G029900, TraesCS2B02G120600, TraesCS2B02G250900, TraesCS2B02G368600, TraesCS2B02G368700, TraesCS2B02G379400, TraesCS2B02G432700, TraesCS2B02G452400, TraesCS2A02G431100, TraesCS2D02G102800, TraesCS2D02G232800, TraesCS2D02G348500, TraesCS2D02G348600, TraesCS2D02G359100, TraesCS2D02G410700, TraesCS2D02G428900, TraesCS3A02G266300, TraesCS3A02G302600, TraesCS3A02G432500, TraesCS3B02G299800, TraesCS3B02G333800, TraesCS3B02G468400, TraesCS3B02G597300, TraesCS3D02G072400, TraesCS3D02G188800, TraesCS3D02G266400, TraesCS3D02G299200, TraesCS3D02G421100, TraesCS3D02G425800, TraesCS3D02G533400, TraesCS4A02G344500, TraesCS4A02G416600, TraesCS4B02G164300, TraesCS4D02G219000, TraesCS4D02G332700, TraesCS5A02G073100, TraesCS5A02G091800, TraesCS5A02G265900, TraesCS5A02G286700, TraesCS5A02G410000, TraesCS5B02G079400, TraesCS5B02G097800, TraesCS5B02G265600, TraesCS5B02G286000, TraesCS5B02G463100, TraesCS5B02G499700, TraesCS5D02G139300, TraesCS5D02G143400, TraesCS5D02G273900, TraesCS5D02G294400, TraesCS5D02G528900, TraesCS5D02G550200, TraesCS6A02G051700, TraesCS6A02G110100, TraesCS6A02G155300, TraesCS6A02G367300, TraesCS6A02G376700, TraesCS6A02G376900, TraesCS6A02G377000, TraesCS6B02G119000, TraesCS6B02G138400, TraesCS6B02G183400, TraesCS6B02G463100, TraesCS6D02G055700, TraesCS6D02G098500, TraesCS6D02G145200, TraesCS7A02G246500, TraesCS7A02G260500, TraesCS7A02G376600, TraesCS7A02G507200, TraesCS7A02G507700, TraesCS7B02G144900, TraesCS7B02G158500, TraesCS7B02G415600, TraesCS7D02G004000, TraesCS7D02G005400, TraesCS7D02G222800, TraesCS7D02G245200, TraesCS7D02G261500, TraesCS7D02G369500* | *TraesCS1A02G107500, TraesCS1B02G080700, TraesCS2A02G232400, TraesCS2A02G413900, TraesCS2A02G431100, TraesCS2B02G094200, TraesCS2B02G250900, TraesCS2B02G432700, TraesCS2B02G452400, TraesCS2D02G076800, TraesCS2D02G232800, TraesCS2D02G410700, TraesCS2D02G428900, TraesCS3A02G432500, TraesCS3B02G468400, TraesCS3D02G072400, TraesCS3D02G425800, TraesCS4D02G332700, TraesCS5A02G265900, TraesCS5A02G286700, TraesCS5B02G265600, TraesCS5B02G286000, TraesCS5B02G395700, TraesCS5B02G505600, TraesCS5D02G273900, TraesCS5D02G294400, TraesCS6A02G110100, TraesCS6A02G155300, TraesCS6A02G414800, TraesCS6B02G138400, TraesCS6B02G183400, TraesCS6B02G463100, TraesCS6D02G098500, TraesCS6D02G145200, TraesCS7A02G246500, TraesCS7A02G260500, TraesCS7A02G376600, TraesCS7B02G144900, TraesCS7B02G158500, TraesCS7B02G185200, TraesCS7D02G245200, TraesCS7D02G261500, TraesCS6B02G183400* |

The red font represents genes that were predicted simultaneously in both methods
